# Alpiniae oxyphylla fructus extract promotes longevity and stress resistance of *C. elegans via* DAF-16 and SKN-1

**DOI:** 10.3389/fphar.2022.1034515

**Published:** 2022-11-24

**Authors:** Man Xiao, Bocen Chen, Kun Niu, Ziyu Long, Fan Yang, Yiqiang Xie

**Affiliations:** ^1^ Key Laboratory of Biochemistry and Molecular Biology, Hainan Medical University, Haikou, Hainan, China; ^2^ College of Chinese Traditional Medicine, Hainan Medical University, Haikou, Hainan, China; ^3^ Hainan Provincial Key Laboratory for Human Reproductive Medicine and Genetic Research, Hainan Medical University, Haikou, Hainan, China

**Keywords:** *Caenorhabditis elegans* (*c. elegans*), *Alpiniae oxyphylla fructus* (AOF), lifespan, insulin/IGF signaling pathway (IIS), aging

## Abstract

**Background:** *Alpiniae Oxyphylla Fructus* (AOF) is Traditional Chinese medicine and a dietary supplements for centuries, which posseses cardiotonic, neuroprotective, antioxidant, warming the kidney and nourish the spleen, these biological fuction is related to potential anti-aging properties. However, little is known about their effects on aging. This work aimed to investigate the effects of extracts of AOF on longevity and stress resistance in *Caenorhabditis elegans* (*C. elegans*) and the mechanisms that underlie its effects.

**Methods:** Wild-type (WT) strand of *C.elegans* (N2)worms were cultured in growth medium with or without AOF. First, we examined the effects of AOF on lifespan, reproduction and healthspan assay, stress resistance and oxidative analysis, lipofuscin levels. Second, The levels of ROS and MDA, the antioxidant enzyme activities were examined to explore the underlying mechanism of AOF. Finally, the expression of the longevity-related genes were investigated to further understand the AOF’s underlying mechanism.

**Results:** The lifespan of *C. elegans* was prolonged by 23.44% after treatment with high-dose AOF (100 ug/ml). AOF alleviated aging-related declines in *C. elegans* health and enhanced resistance to heat shock. Furthermore, AOF decreased reactive oxygen species and malondialdehyde, increased the activities of superoxide dismutase and catalase, and reduced accumulation of fat. AOF upregulated the expression of sod-3, gst-4, daf-16, and skn-1 but downregulated the expression of daf-2 and age-1 and accelerated the translocation of DAF-16 into the nucleus. The extended lifespan induced by AOF was reversed in *daf-16(mu86)* and *skn-1(zu135*) mutants, indicating that this gene is involved in AOF-regulated longevity.

**Conclusion:** Our findings demonstrated that AOF extends lifespan and healthspan and enhances stress *via* boosting the activity of the antioxidant enzyme and controlling the expression of genes associated with insulin/IGF signaling and SKN-1 pathways. As a result, this work suggested AOF as a possible candidate to reduce the signs of aging by activating and inhibiting target genes.

## 1 Introduction

Over the last 20 years, the percentage of people over age 65 has increased globally. Aging presents both social problems and physiological problems, including declines in stress resistance, metabolic rate, and immune function (Fuellen G et al., 2019). Aging cells exhibit mitochondrial dysfunction, genomic instability, loss of proteostasis, stem cell exhaustion, cellular senescence, and altered intercellular communication (Jing L et al., 2017). Treatments to slow or reverse the changes associated with aging are urgently needed.

Along with the development of modern medicine, there has been increasing interest in traditional herbal medicines that delay the aging process and prevent aging-related diseases (Ke-Ji et al., 2015; Zhang Q et al., 2015; Wan et al., 2020). For example, Yizhi Xingnao decoction and Bu-Shen-Yi-Zhi prescription (BSYZ) have achieved good therapeutic effects in patients with Alzheimer’s disease and senile plaques. Traditional Chinese medicine theory holds that kidney reinforcing, promoting blood circulation and removing blood stasis, and activating Qi flow through energy meridians and invigorating the spleen have the function of delaying aging.

*Alpiniae Oxyphylla Fructuse* (AOF, Yizhi in Chinese), a well-known edible medicinal herb (Wang et al., 2015), is the mature and dried fruit of Alpinia oxyphylla Miq, which is a perennial plant of the Zingiberaceae family. AOF is oval to nearly round, and when the color changes from brown to red, it is harvested and dried in the shade with ventilation. It is one of the four famous medicines in southern China. The functions of AOF include that nourishing the spleen prevents diarrhea and reduces spittle, reinforcing the kidney essence to decrease urination, as well as brain tonic promote the circulation of Qi and blood to recovery of function (Jiang W et al., 2022). As the same time, pharmacological research find AOF plays critical roles in various diseases. For example, in the treatment of Alzheimer’s disease (AD), AOF can prevent the synthesis of amyloid-β (Aβ) and aberrant phosphorylation of tau protein, which in turn affects the functions of anti-oxidation, anti-inflammatory, suppressing apoptosis, protecting of the nervous system, etc (Li J et al., 2020). In rat models of chronic intermittent hypoxia (CIH)-induced enuresis, AOF relieves enuresis by reducing oxidative stress and the expression of the purinergic P2X3 receptor, muscarinic M3 receptor, and increasing the expression ß3 adrenergic receptor (Su M S et al., 2020). In HFD-induced MAFLD mice, AOF regulates hepatocyte glucose and lipid metabolism by AMPK activation and play anti-inflammatory effects by MAPK signaling inhibition (Yong Z et al., 2022). By modifying the composition of the gut microbiota, AOF treatment lowered blood glucose levels and dramatically improved kidney pathological damage in T2DM mice (Xie Y et al., 2018). In colorectal cancer cells AOF displayed antioxidant and antiproliferative activity *via* cyclin D1suppression and NAG-1 increasing (Yoo E et al., 2020). Furthermore, AOF play a major role protects in senescent cardiomyoblasts and cardiac aging rats, AoF treatments notably showed increased DNA replication,decrease S phase mediated DNA replication, attenuate D-gal and P21 (Chang Y M et al., 2021). Moreover, through down-regulation of collagen-related accumulation/degradative pathways and further maintenance of collagen homoeostasis, AOF therapy reduced the risk for cardiac fibrosis (Chang Y M et al., 2019). In summary, the results of these researches showed AOF has therapeutic potency and healthcare functions in a number of illnesses.

The nematode *Caenorhabditis elegans (C.elegans)* is a model organism used widely in research, including the response to aging and stress (Moreira CA et al., 2019). It is an ideal research model because of its short generation time, simple growth conditions, simple anatomy, and genes that are homologous to human genes (Hastings J et al., 2019). The insulin/insulin-like growth factor-1 (IGF-1) signaling (IIS) pathway and target of rapamycin (TOR) pathway are well-studied signaling pathways involved in aging. The evolutionarily conserved IIS pathway is commonly used to screen for anti-aging drugs (Saikia and Bordoloi, 2019) because the dysregulation of genes in the IIS pathway contributes to the development of aging-related diseases in humans. DAF-16 and SKN-1 are conserved downstream transcription factors in the IIS pathway that promote longevity in *C.elegans* (Duangjan C, et al., 2019). Previous studies have shown that DAF-16/FOXO and SKN-1/Nrf-2 signaling pathways enhance the anti-oxidative stress response to prolong life (Wan Q L,et al., 2020).

Although AOF is recognized as an anti-aging drug in the medical literature, there has been little research on the molecular mechanisms that explain how AOF extends healthspan or lifespan. We investigated the anti-aging effects of AOF in *C.elegans* to determine the biochemical activities and pathways that explain the extended lifespan and healthspan and stress-resistance effects of AOF. Our results provide new evidence on the molecular mechanisms that underlie AOF effects and its promise as a dietary supplement to increase healthspan and delay or prevent age-related diseases.

## 2 Materials and methods

### 2.1 Materials and reagents

5-Fluoro-2′-deoxyuridine (FUDR, ≥99.9%) and the ROS Green Fluorescence Detection Kit were purchased from Sigma Aldrich Chemical Co. (St. Louis, MO, United States). Methyl viologen dichloride (paraquat, 98%) was provided by Aladdin Reagent Co. (Shanghai, China). All other chemicals and solvents were of analytical grade or above.

The *C.elegans* strains and *Escherichia coli* OP50 used in the study were provided by Luo Huairong’s research team, Kunming Institute of Botany. The *C.elegans* strains included the wild-type *C.elegans* N2 (Bristol), *TJ356 (daf-16:GFP), daf-16(mu86) I, daf-2(e1370), age-1(hx546), skn-1(zu135) IV,LD1 (skn-1:GFP), CL2166(gst-4:GFP), CF1553 (sod-3:GFP), hsf-1::GFP(OG532)* strains. All strains were grown and maintained at 20°C on solid nematode growth medium (NGM) plates.Among them, the strains were all from LUO Huairong’s and Shao Zhiyong’s research teams.

Metabolite profiles were generated through ultrahigh-performance liquid chromatograph Q extractive mass spectrometry (UHPLC-QE-MS) by Shanghai Biotree Biomedical Technology Co., LTD.

### 2.2 AOF

*Alpinia oxyphylla Miq* (AOF) (batch no. C026181218) was purchased from Sichuan Guoqiang Traditional Chinese Medicine Yinpian Co., LTD. According to the “Chinese Pharmacopoeia” 2015 edition of the identified in compliance with the provisions as genuine.We will resuspended *Escherichia coli* OP50 with AOF diluted with sterile water of corresponding concentration, and add *Escherichia coli* OP50 containing 100 ug/mlAOF as food drop on NGM plate as the way of administration.

#### 2.3 LC-MS/MS untargeted metabolomics analysis of AOF

An AOF sample of 100 mg of was added to 500 μL of extraction solution containing 1 μg/ml of internal standard, homogenized at 45 Hz for 4 min, and sonicated for 1 h in an ice-water bath. After 1 h at −20°C, the samples were centrifuged at 12,000 rpm (RCF = 13,800 × g, R = 8.6 cm) for 15 min at 4°C. The supernatant was transferred to a 2-ml tube for untargeted LC-MS/MS UHPLC-QTOF-MS analysis (Biotree Biotechnology, Shanghai, China).

#### 2.4 Pathway enrichment analysis (KEGG)

KEGG was selected for pathway analysis, and significant enrichment pathway was obtained under *p < 0.05* after enrichment results were presented. The enrichment results were drawn into bubble diagram.

#### 2.5 Lifespan assay

*C.elegans* was age-synchronized by the sodium perchlorate lysis method. The hermaphrodite *C.elegans* in the spawning period was washed from the NGM plate with sterile M9 solution, and the nematode-containing solution was placed in 1 ml of M9 solution in a microcentrifuge tube. A solution of 4.5 ml 0.5 M NaOH and 0.5 ml 5% NaOCl was added to the 5-ml lysate, the tube was inverted and mixed for 3–5 min, and centrifuged at 3,000 rpm for 1 min. The supernatant was discarded, and the precipitate was washed 2–3 times with sterile M9 solution and then transferred to the NGM plate containing *E. coli* OP50 and 50 μM FUdR for culture at 20°C. About 48 h later, the fertilized eggs of *C.elegans* developed to the L4 stage and completed the synchronization.

To determine lifespan, the synchronized L4 *C.elegans* were placed in the medium for each group, with 50 *C.elegans* per board and 150 *C.elegans* per group, and cultured at 20°C. *C.elegans* were transferred to a new solid medium every 2 days after they developed into reproductive adults. Nematodes that failed to respond to touches to their head and tail with a platinum wire for 30 s were judged to be dead. *C.elegans* was observed under a stereomicroscope to determine survival and death. *C.elegans* that climbed out of the culture plate were removed. The experiment was repeated three times.

#### 2.6 Reproduction assay

The synchronized *C.elegans* were divided into the control group and the 100 μg/ml AOF group. Five animals from each group were transferred individually to NGM plates on the first day of the reproductive ability test. Subsequently, the *C.elegans* were transferred daily to new NGM plates to lay eggs until reproductive capacity disappeared. The number of offspring on the first, third, fifth, and seventh days after spawning was recorded. The reproductive period of *C.elegans* was generally 7 days, and the total number of offspring was the sum of the daily offspring. Experiments are the average of three independent trials.

#### 2.7 Healthspan assays

##### 2.7.1 Pharyngeal pumping assay

The pharyngeal pumping assay was conducted according to the classical method (Onken B et al. .,2010). Age-synchronized young adult worms were treated with 100 mg/ml AOF and the control group with *E. coli* OP50 for 10 days, and the pharyngeal pumping rate was measured under a dissecting microscope for 30 s. The experiment was repeated three times.

##### 2.7.2 Locomotion assay-body bending

Age-synchronized young adult worms were treated with 100 mg/ml AOF and the control group with *E. coli* OP50 on the 14th day of adulthood, and the motility classes were evaluated according to the method of Golden et al. (T. R. Golden et al., 2008), with little modification. The movement assay was repeated three times.

##### 2.7.3 Head swings assay

Age-synchronized late L4 larvae or young adult worms were transferred to plates with 100 mg/ml AOF and the control group with *E. coli* OP50 and cultured for 10°days at 20°C. Before the head swings assay, worms were gently transferred to a plate with a drop of M9 buffer and left to acclimatize for 15°s at room temperature. A positive response was scored if the worm stopped forward movement and also initiated a reversal that lasted at least half a head swing, as observed under a stereomicroscope. The assay was repeated at least three times.

#### 2.8 Stress resistance assays

##### 2.8.1 Thermal shock assay

Age-synchronized young adult worms were treated with 100 mg/ml AOF and the control group with *E. coli* OP50 on the 10th day of adulthood at 20°C. The treated nematodes were incubated daily for two hours at 35°C, and dead worms were counted every day. The assay was repeated at least three times.We have tested the transcriptional levels of hsf-1 hsp-70 and testing of the transgenic strains of *hsf- 1: GFP (OG5335)* after AOF treatment.

##### 2.8.2 Paraquat-induced oxidative stress assay

Age-synchronized young adult worms were treated with 100 mg/ml AOF and the control group with *E. coli* OP50 on the 10th day of adulthood at 20°C. The treated worms were incubated with shaking for 2 h in 1 ml M9 buffer containing 125 mM paraquat, washed with M9 buffer three times, transferred to NGM plates seeded with *E. coli* OP50, and incubated for 24 h at 20°C. The surviving animals were counted until all died.

##### 2.8.3 Lipofuscin level

The synchronized larvae, grown for 16–20 h, were divided into the 100 mg/ml AOF and the control groups and grown for an additional 10 days. The oil red O stock solution in the kit (Sigma-Aldrich, St. Louis, MO, United States) was prepared daily in a ratio of three parts of oil red O stock solution to 2 parts sterile ddH2O and filtered through a microporous filter. The nematodes were treated with the fixative solution provided by the kit for 15–20 min, washed at least three times to remove the fixative, stained with the oil red O dye at room temperature for 20 min, and washed until there was no dye residue. Specimens were photographed under a microscope on slides.The L4 adult worms were treated with 100 mg/ml, while control plates were treated with 50 μM FUDR for 10 days. All trials were repeated three times.

#### 2.9 Reactive oxygen species (ROS), malondialdehyde (MDA), superoxide dismutase (SOD), and catalase (CAT) in the nematodes

A kit was used to measure ROS levels by fluorescence microscopy in the nematodes after AOF treatment [18]. The activities of SOD and CAT and the MDA content in the supernatant of 2000 mechanically-ground nematodes, treated with 100 mg/ml AOF, were determined using commercial kits according to the manufacturer’s instructions (JIANCHENG, Nanjing, China). All values were normalized to protein levels determined using a commercial BCA kit (Beyotime Biotechnology).

#### 2.10 Reverse transcriptase-quantitative polymerase chain reaction (RT-qPCR) assay

cDNA was synthesized from total RNA (Promega, Beijing, China) isolated from about 2000 worms (treated for 10 days) by reverse transcriptase using the Hifair^®^Ⅱ1st Strand cDNA Synthesis Kit (Yeason Biotechnology, Shanghai, China). After qPCR, using the Hieff^®^ qPCR SYBR Green Master Mix (Low Rox Plus) (Yeason Biotechnology, Shanghai, China), gene expression levels were expressed as 2^−ΔΔCT^ using cdc-42 as the reference gene (T. R. Golden et al.,2008). The primers used for RT-qPCR are listed in Supplementary Table S1.

#### 2.11 The fluorescent observation of the transgenic strain *skn-1:GFP, daf-16: GFP, hsf-1: GFP*

The change of fluorescence intensity was observed under a fluorescence microscope.

#### 2.12 Statistical analysis

The data were expressed as mean ± SD. The results were analyzed using GraphPad Prism 6.0 and SPSS 20.0 software. The above experimental results are obtained by more than three independent repetitive operations, and all experimental data are expressed as mean ± standard deviation. The results of survival curve experiment were analyzed by “log-rank test”. The statistical test level is α = 0.05, and *p* < 0.05 indicated that the difference is statistically significant. *p* < 0.001 and below indicated that the difference is statistically significant.

## 3 Results

### 3.1 Chemical composition of AOF

**FIGURE 1:**
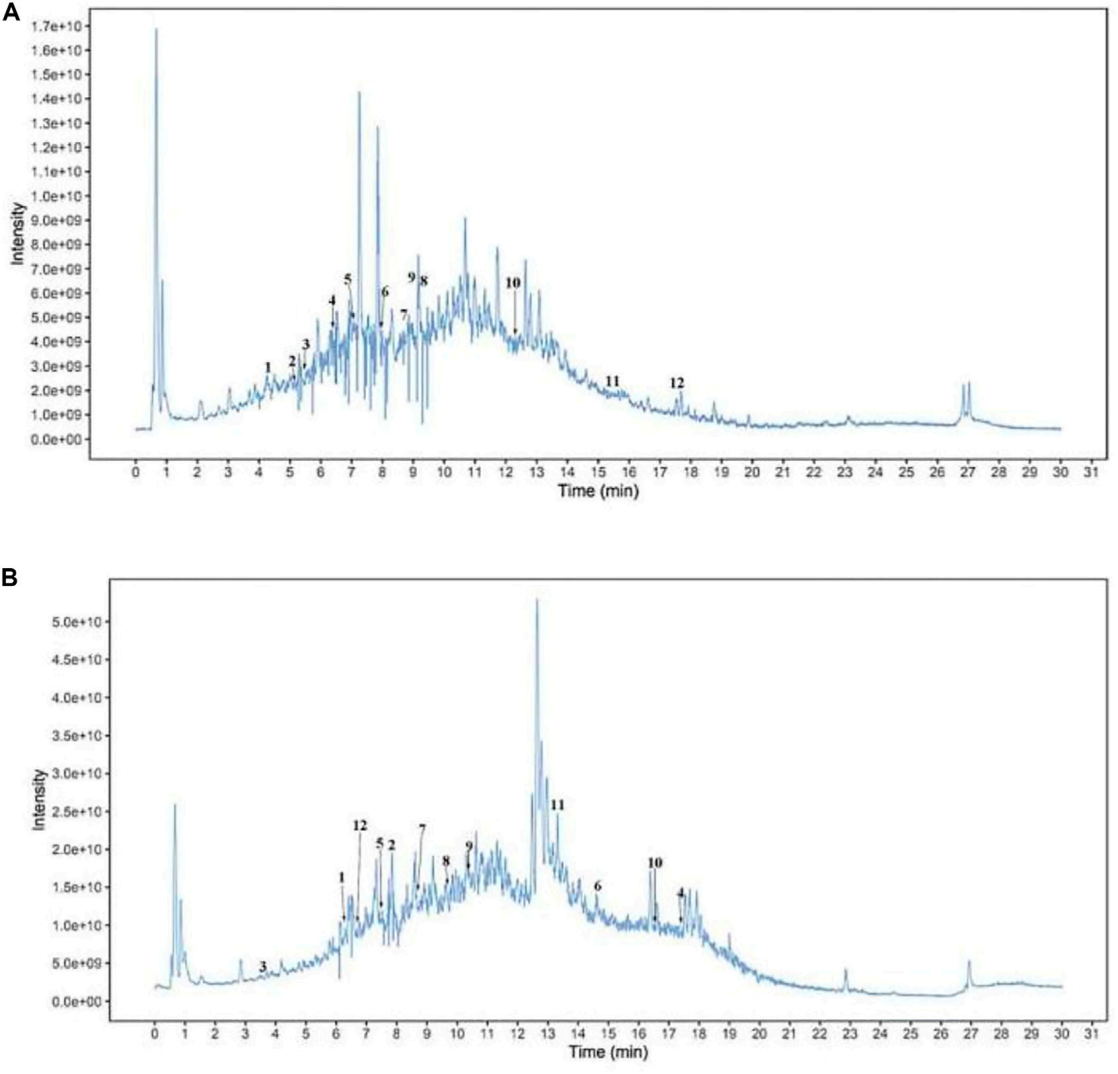
Metabolites in AOF. Total ion current pattern of **(A)** positive ions and **(B)** negative ions detected by UHPLC-QTOF-MS of AOF. 1: citrate; 2: lauryl sulfate; 3:Benzene-1,2,4-triol; 4: 5,9-dihydroxy-5,7,7-trimethyl-4,5a,6,8,8a, 9-hexahydro-1H-azuleno [5,6-c]furan-3-one; 5: isorhamnetin; 6: D-gluconic acid; 7: 6-gingerol; 8: D-(+)-malic acid; 9: (+)-nootkatone; 10: alpha-cyperone; 11: choline [M]+; 12: germacrone.

Untargeted LC-MS/MS UHPLC-QTOF-MS analysis (Biotree Biotechnology, Shanghai, China) of AOF revealed large peaks for citrate, lauryl sulfate, 6-gingerol, and several other compounds (Figure 1A, Figure 1B, and Table 1).

**TABLE 1:** A partial metabolite of AOF.

Name	Class
Citrate	Organic acids and derivatives
lauryl sulfate	Miscellaneous
5,9-dihydroxy-5,7,7-trimethyl-4,5a,6,8,8a,9-hexahydro-1H-azuleno [5,6-c]furan-3-one	Terpenoids
D-gluconic acid	Organic acids and derivatives
6-gingerol	Phenols
D-(+)-malic acid	Hydroxy acids and derivatives
(+)-nootkatone	Terpenoids
alpha-cyperone	Terpenoids
choline [M]+	Organonitrogen compounds
Germacrone	Sesquiterpenoids

### 3.2 KEGG pathway analysis of AOF metabolites

**FIGURE 2:**
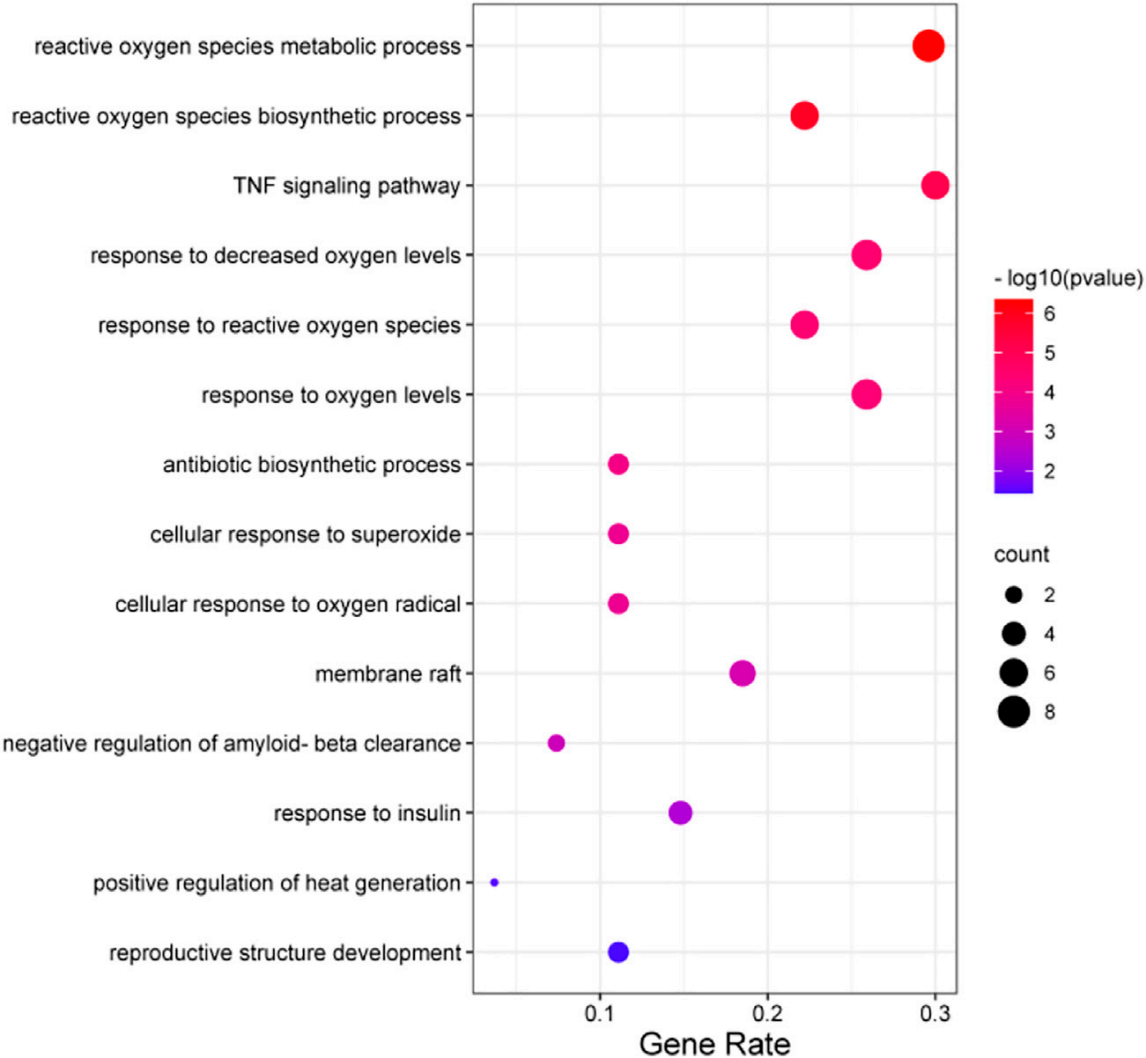
KEGG pathway analysis of AOF metabolites.

KEGG pathway enrichment analysis of AOF metabolites shown in Table 1 revealed pathways mainly involved in ROS metabolic processes, tumor necrosis factor (TNF) signaling pathway, and antibiotic biosynthetic process (Figure 2).

### 3.3 AOF increased the lifespan of *C.elegans*

**FIGURE 3:**
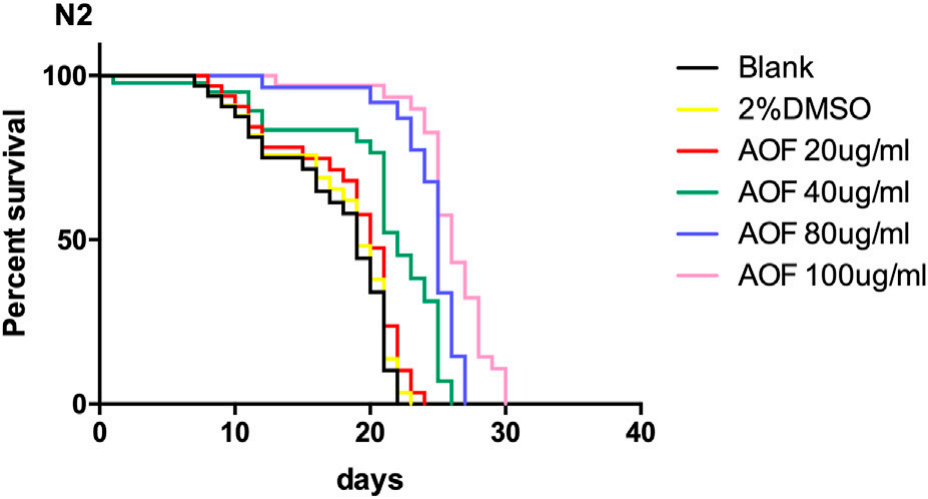
Effects of AOF extract concentration on the survival of wild-type *C.elegans* N2. Data are the mean ± SD (*n* = 3).

The mean and maximum life expectancy values for the AOF-treated *C.elegans* group increased in a dose-dependent manner; thus, AOF significantly prolonged the lifespan of *C.elegans*. The mean life expectancy of *C.elegans* treated with 100 mg/ml AOF was 23.44% longer than the control groups. Therefore, AOF delayed senescence and prolonged the lifespan of wild-type N2 nematodes (Figure 3).

### 3.4 Fertility of AOF-treated *C.elegans*

**FIGURE 4:**
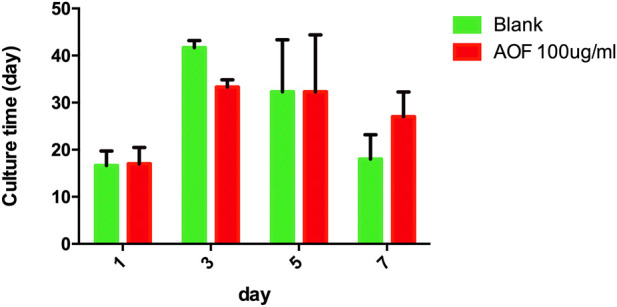
Effect of AOF treatment on reproduction in *C.elegans*. Data are the mean ± SD (*n* = 3),*p* > 0.05 for all AOF groups vs Blank groups.

To determine whether increased lifespan affected fertility, we measured the fertility in the AOF-treated group vs the control. There was no significant difference in the number of offspring per day between the two groups *(p > 0.05*, Figure 4), indicating that the AOF extract had no effect on reproduction in *C.elegans*.

### 3.5 Effects of AOF on the motility of *C.elegans*

**FIGURE 5:**
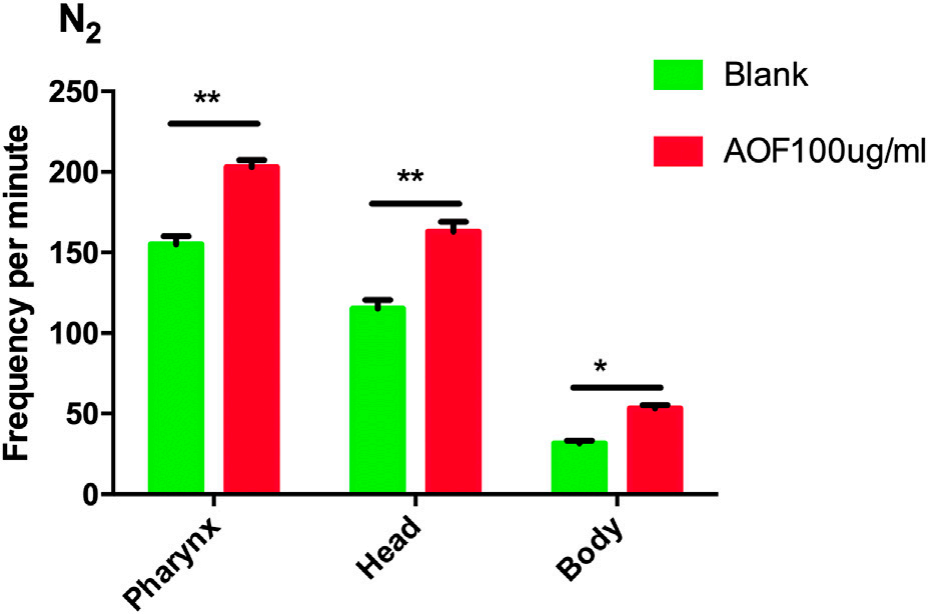
Effect of AOF treatment on the motility of wild-type *C.elegans* N2. Data are the mean ± SD (*n* = 3), *p < 0.05, **p < 0.01.

To determine whether there was an improvement in general health associated with AOF treatment, we measured locomotion and the pumping of pharyngeal muscles, which are similar to the heartbeat of humans. AOF significantly increased the pumping rate by 33.78% in 30 s compared to the control group. Motor behavior can be assessed by observing the frequency of head swings and the degree of body bending. The AOF-treated group significantly increased the frequency of head swings by 27.19% compared to the control group, and body bending improved by 19.23%. These parameters showed that AOF improved healthspan and decreased the age-related decline related to these phenotypes (Figure 5).

### 3.6 Effect of AOF on stress resistance in *C.elegans*

**FIGURE 6:**
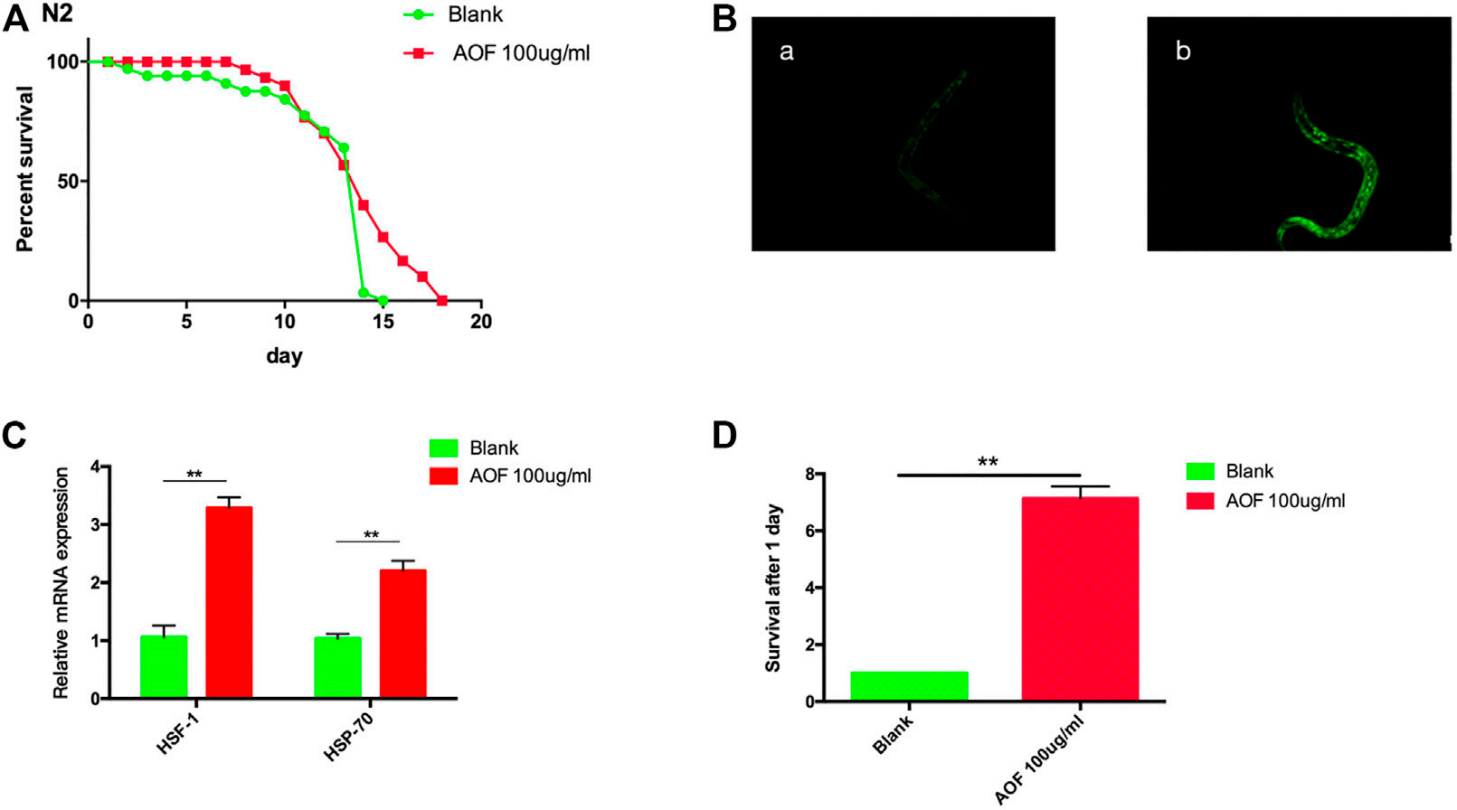
Effect of AOF treatment on **(C)** *elegans* N2. **(A)** heat-shock survival at 35°C, and **(B)** a representative image of the nuclear HSF-1:GFP fraction in **(A)**, Blank group and **(B)**, 100 μg/ml AOF group,and **(C)** the transcriptional levels of hsf-1、hsp-70, and **(D)** paraquat-induced oxidative stress survival. Data are the mean ± SD (*n* = 3). **p < 0.01.

Increased lifespan is often accompanied by increased stress resistance, and lifespan is positively correlated with the ability to resist stresses. Therefore, we measured the response of *C.elegans* at the L4 stage to abiotic heat-shock stress and paraquat-induced oxidative stress after treatment with 100 mg/ml AOF for every days. Thermotolerance was enhanced by AOF treatment. The *C.elegans* pretreated with 100 μg/ml AOF were then exposed to heat-stress (35°C) for 48 h and monitored every day death. The survival time for wild-type nematodes increased by 27.3% in the AOF treated group compared with the Blank group (Figure 6A). We found a significant increase in nuclear translocation of DAF-16:GFP in nematodes treated with AOF compared to the control (Figure 6B).We have tested the transcriptional levels of hsf-1 hsp-70. At the same time, the expression of these genes increased significantly in AOF-treated nematodes vs the Blank group. (Figure 6C).

In the paraquat-induced oxidative stress assay, nematodes were placed in tubes containing 125 mM paraquat for 2.5 h, and the number of surviving *C.elegans* was counted in AOF group was 7.12 times higher than that in Blank group had 24 h later (Figure 6D). These data suggest that pretreatment with AOF induced resistance against damage from heat stress and oxidative stress.

### 3.7 Fat observation of intestinal lipid deposition

**FIGURE 7:**
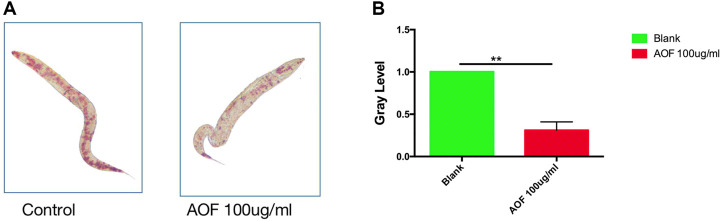
Effect of AOF treatment on lipofuscin accumulation in *C.elegans* **(A–B)**. Data are the mean ± SD (*n* = 3). **p < 0.01.

Increased longevity may be related to fat metabolism; therefore, we determined whether AOF reduced fat accumulation *in vivo*. Using oil red O staining to measure fat accumulation in the intestinal tract, we determined that the AOF-treated worms had significantly less fat than the control group (Figure 7A), and comparisons of gray levels showed a 61.2% reduction in fat after 24 h of AOF treatment (Figure 7B).

### 3.8 AOF diminished ROS and increased antioxidant enzyme activities

**FIGURE 8:**
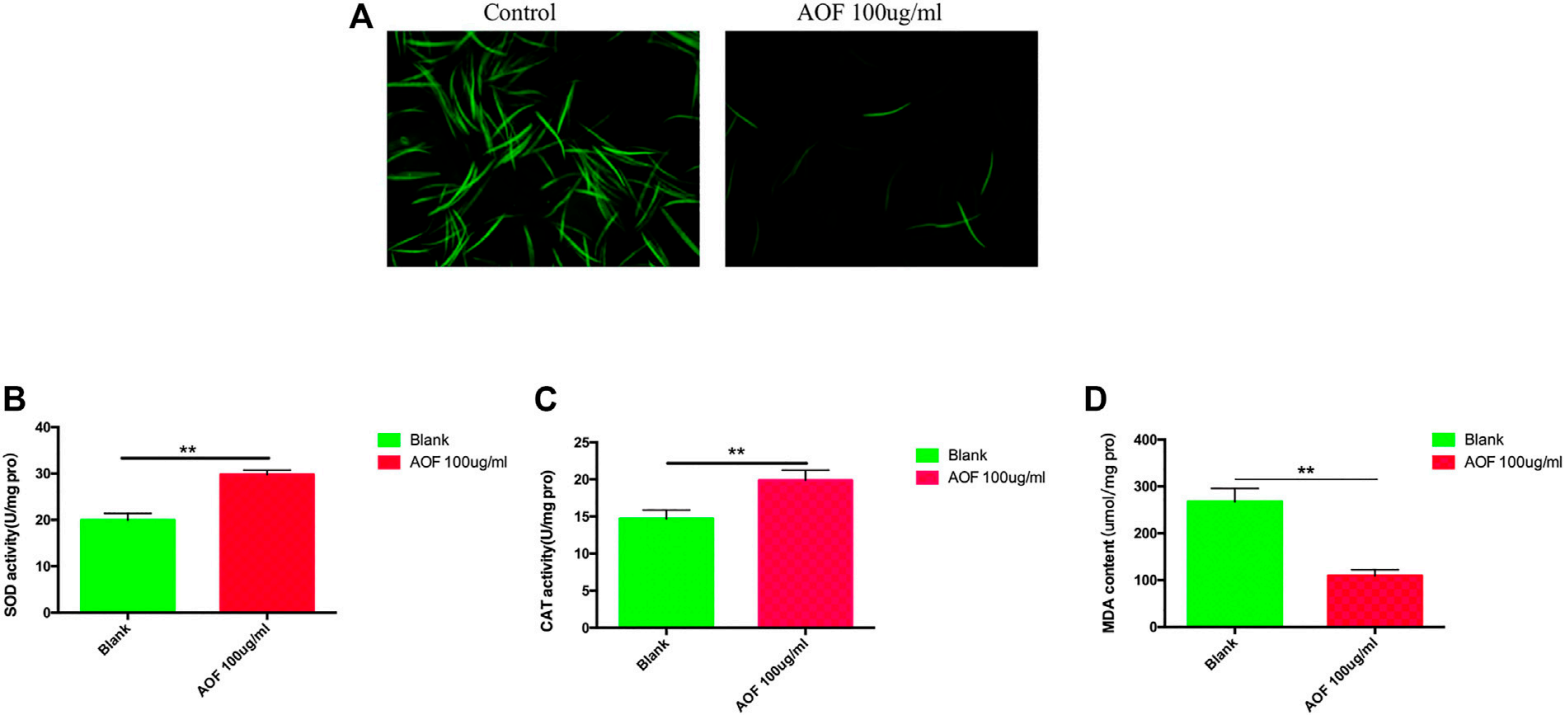
Antioxidant effects of AOF treatment of **(C)** *elegans*. **(A)** Effect of AOF on ROS-mediated fluorescence. **(B–D)** Effect of AOF on the activities of antioxidant enzymes **(B)** SOD and **(C)** CAT, and **(D)** the level of MDA. Pro = protein. Data are the mean ± SD (*n* = 3). **p < 0.01.

The levels of ROS, which are by-products of metabolism, were determined by DCFH-DA using fluorescence microscopy. ROS levels were greatly reduced in the AOF group compared with the control group (Figure 8A). SOD and the MDA content are important indexes of oxidative stress. SOD activity increased significantly by 46.4% in the AOF-treated group compared with the control group (Figure 8B), and the results were similar for CAT activity with a 33.4% increase (Figure 8C). The MDA content was notably reduced by 62.1% in the AOF-treated group vs the control group (Figure 8D). AOF showed its potential to reduce ROS and MDA and enhance the activities of SOD and CAT.

### 3.9 AOF regulated the insulin/IGF-1 signaling (IIS) pathway

**FIGURE 9:**
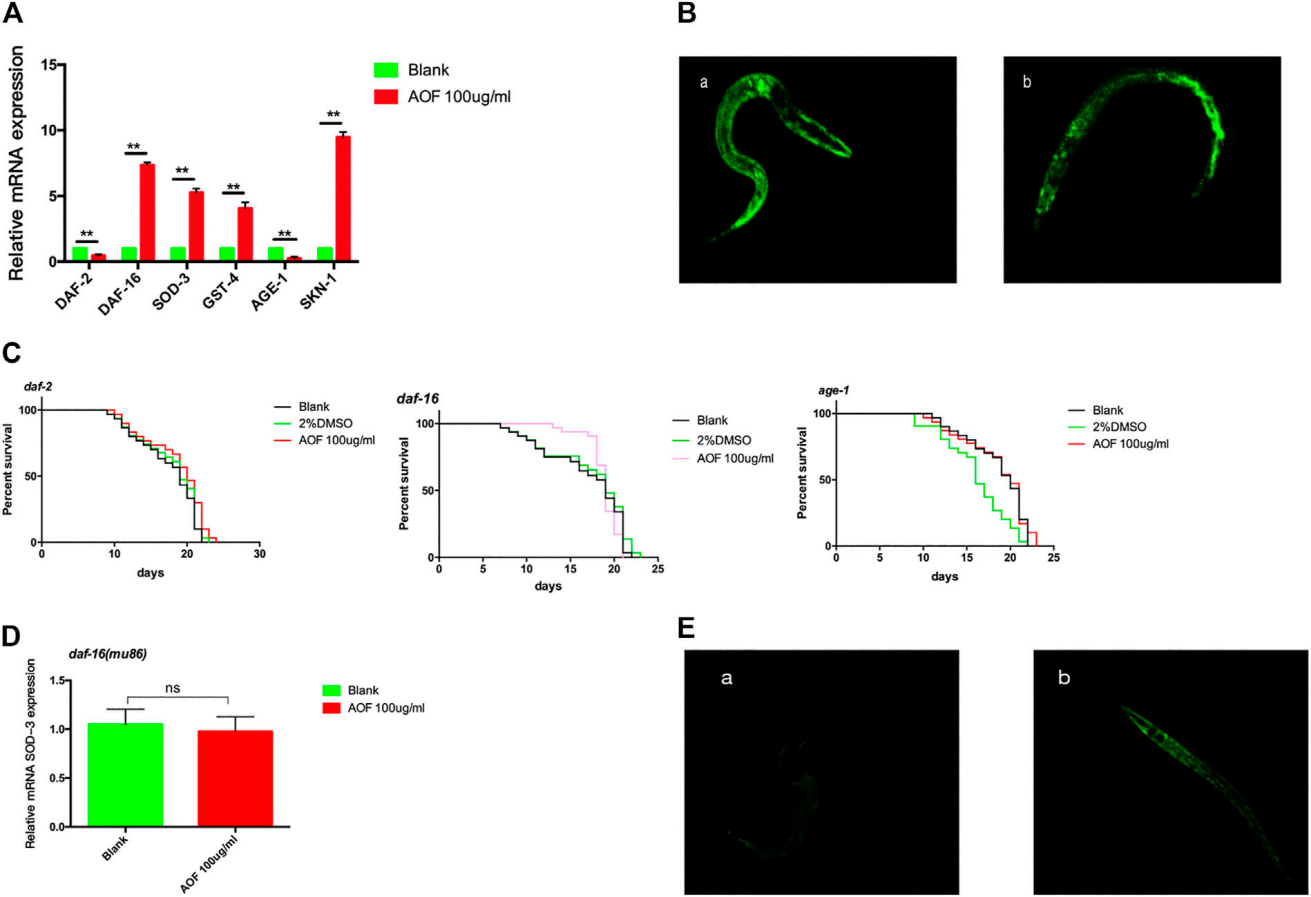
The role of the DAF-2-DAF-16 signaling pathway in the anti-aging effects of AOF. **(A)** Relative expression of aging-related genes in **(C)** *elegans*. **(B)** A representative image of DAF-16:GFP expression in **(A)**, control group and **(B)**, 100 μg/ml AOF group. **(C)** Effect of AOF on the lifespan of daf-2, daf-16, or age-1 mutant worms. **(D)** The relative SOD-3 mRNA expression in daf-16 mutants. **(E)** Fluorescence intensity of sod-3:GFP after AOF in **(A)**, control group and **(B)**, 100 μg/ml AOF group.Data are the mean ± SD (n = 3). **p < 0.01.

Insulin/insulin-like growth factor-1 signaling (IIS), which is a conserved pathway in invertebrates and vertebrates, was the first pathway shown to be involved in the regulation of aging in *C.elegans*. To study the anti-aging mechanisms of AOF, we measured the expression of the daf-16, sod-3, gst-4, and age-1 genes that are related to the IIS pathway. We determined that the expression of these genes increased significantly in AOF-treated nematodes vs the control, but the expression of daf-2 decreased (Figure 9A), suggesting that the IIS pathway contributes to the beneficial effects of AOF on lifespan.

DAF-16, which activates the expression of proteins associated with resistance to stress responses, shuttles from the cytoplasm to the nucleus to integrate multiple signaling pathways. We found a significant increase in nuclear translocation of DAF-16:GFP in nematodes treated with AOF compared to the control (Figure 9B). To better understand the mechanism(s) through which AOF extends the lifespan of *C.elegans via* the DAF-2-DAF-16 signaling pathway, we treated daf-2, daf-16, or age-1 mutant worms with AOF for 5 days at the start of the young adult stage and determined their lifespan. In each of these mutants, the mean lifespan (21.44 ± 0.57 days for daf-2, 21.06 ± 0.37 days for daf-16, and 22.10 ± 0.76 days for age-1) did not increase significantly after treatment with AOF (Figure 9C).We have tested the transcriptional levels of the SOD-3 mRNA level in the daf-16 mutants after AOF treatment, It was found that there was no significant difference in the relative expression of SOD-3 after AOF (Figure 9D).We further detected the fluorescence expression of sod-3:GFP, and found that the fluorescence expression was significantly increased after AOF (Figure 9E).

### 3.10 SKN-1 activation by AOF

**FIGURE 10:**
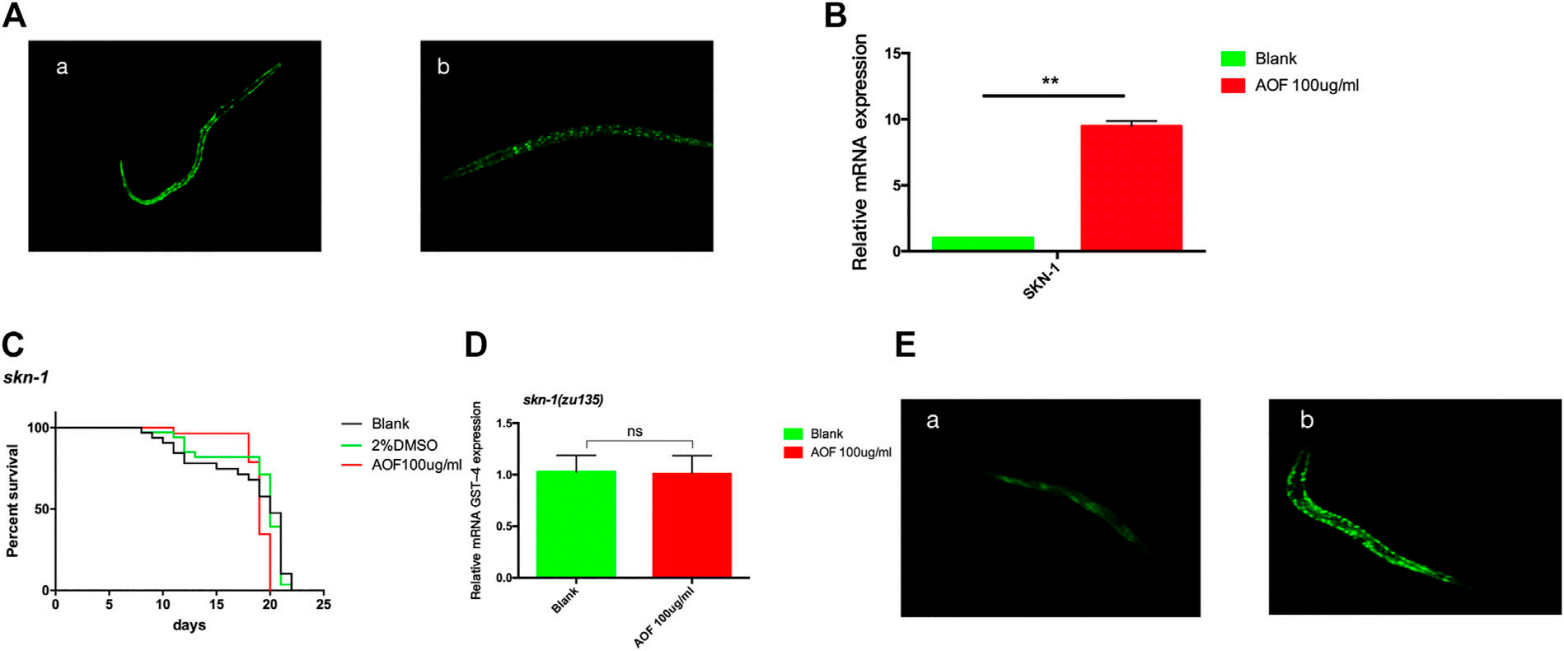
The role of SKN-1 in the anti-aging effects of AOF. **(A)** A representative image of SKN-1:GFP in strain *skn-1(zu135)* for **(A)**, control group and **(B)**, 100 μg/ml AOF group. **(B)** The expression of skn-1 in the AOF treatment group vs control group. **(C)** Effect of AOF on the lifespan of the *skn-1(zu135)* mutant. **(D)** The relative GST-4 mRNA expression in *skn-1(zu135)* mutants. **(E)** Fluorescence intensity of GST-4:GFP after AOF in **(A)**, control group and **(B)**, 100 μg/ml AOF group.Data are the mean ± SD (*n* = 3). **p < 0.01.

Transcription factor SKN-1 promotes resistance to several aging-related oxidative stresses, and the SKN-1 pathway in *C.elegans* is homologous to the Nrf2 pathway in mammals. SKN-1 also affects longevity independent of DAF-16/FOXO. Similar to Nrf2, SKN-1 regulates nuclear localization and transcriptional activation, thereby maintaining redox homeostasis. We used SKN-1:GFP and *skn-1(zu135)* strains to study the role of the SKN-1 pathway in the longevity of *C.elegans* exposed to AOF. The nuclear SKN-1:GFP fraction increased in AOF-treated nematodes compared to the control (Figure 10A), suggesting post-translational regulation of SKN-1 by AOF. Also, skn-1 was upregulated 8.23-fold in the AOF group compared to the control (Figure 10B). Similarly, after AOF treatment, there was no significant increase in the mean lifespan of the *skn-1(zu135)* mutant (Figure 10C), indicating that the SKN-1 signaling pathway also plays a key role in the AOF-mediated increase in the lifespan of *C.elegans*.We have tested the transcriptional levels of the GST-4 mRNA level in the *skn-1(zu135)* mutants after AOF treatment, It was found that there was no significant difference in the relative expression of GST-4 after AOF (Figure 10D).We further detected the fluorescence expression of gst-4:GFP, and found that the fluorescence expression was significantly increased after AOF (Figure 10E).

## 4 Discussion

Aging is the greatest risk factor for human diseases, and there is increasing interest in understanding age-related diseases. Identifying and characterizing compounds and extracts derived from edible plants that can delay aging is important in research on aging. The fruit of *Alpinia oxyphylla Miq*, mainly produced in Hainan province, is one of the four major southern medicines in China. We have shown previously that AOF has antioxidant and anti-inflammatory activity and reduces kidney injury resulting from high blood glucose levels *in vitro* and *in vivo* (Du et al., 2017). Our study here broadens our understanding of the biological effects of AOF phytochemicals. In the study, we demonstrated that it prolongs the lifespan and healthspan of *C.elegans* in a concentration-dependent manner.

Because AOF comprises multiple therapeutic agents that may function *via* diverse mechanisms, we used the simple but efficient UHPLC-QTOF-MS method to analyze the chemoprofile of AOF and found that terpenoids and organic acids are common in AOF. Terpenoids, a structurally diverse group, are secondary metabolites widely distributed in nature, endowed with a wide range of biological activities that have antibacterial, anti-inflammatory, antitumoral, or neuroprotective effects. Nootkatone is a sesquiterpene derivative. Studies have shown that Nootkatone can has an anti-inflammatory activity *via* inhibition of TLR 4—NF-κB-NLRP3 signaling pathway (Qi Y et al., 2019; Bezerra et al., 2020; Jha A K et al., 2022). In the same way, atalpha-cyperone also as a kind of terpenoids. Some data demonstrated the atalpha-cyperone significant ameliorates IL-1β-induced inflammation *via* NF-κB and MAPKs signaling pathways, which achieve the purpose of treating OA. (Zhang H et al., 2021).The study found that atalpha-cyperone as a fungicidal antifungal agent that can synergize with the activity of a fluconazole antifungal drug that can be used in combination antifungal therapy and some results showed that atalpha-cyperone exerted neuroprotective effects against H_2_O_2_-induced cell apoptosis in SH-SY5Y cells. (Horn and Vediyappan, 2021). As an Organic acids, it has been reported that gluconic acid can also be considered a marker of oxidative stress and some results indicated that supplementation with glucose oxidase could increase antioxidant capacity, immune function and improve the intestinal microbiota composition of broilers. (Ament Z et al., 2021; Wang Y et al., 2022). At the same time, other research groups have found that germacrone could repair mitochondrial damage and strengthen antioxidant capacity to inhibit the production of oxidation products, thereby preventing podocyte apoptosis and relieving DN (Wang Y et al., 2021). These characteristics contribute to their therapeutic value and may increase life expectancy. These compounds have attracted attention for the bioactivities associated with them by KEGG, such as ROS metabolic process, the TNF signaling pathway, and the antibiotic biosynthetic process.

In *C.elegans*, aging is a response to external stimuli such as ambient temperature or paraquat-induced oxidative stress (L González-Cofrade et al., 2019). Our results show the AOF enhancement of resistance to stress correlates positively with increased longevity. Aging in *C.elegans* is also accompanied by changes in crawling behavior and pharyngeal pumping. AOF provided better protection to *C.elegans* from declines in pumping and bending as aging progressed. Collectively, these data indicate that AOF improved not only the lifespan but also the healthspan of wild-type worms and suggests that it has great anti-aging potential.

Aging-related dysfunction due to cellular senescence likely results from the accumulation of oxidative damage to cellular constituents, such as the inflammatory infiltration of neutrophils, increased secretion of proteases, and production of a large number of oxidative intermediates (Han et al., 2020). To identify the anti-oxidative mechanisms by which AOF exerts anti-aging effects, we measured the ROS levels, MDA, and the antioxidant enzyme activities of SOD and CAT in *C. elegans* and found that AOF significantly attenuated the generation of intracellular ROS and MDA and increased SOD and CAT. This suggested that the antioxidant activity of AOF contributes to its anti-aging effect, thereby increasing the ability of cells to prevent or repair oxidative damage, extending lifespan.

In *C. elegans* with reduced IIS pathway activity are resistant to heat and oxidative stress, indicating that an increased ability to prevent or repair oxidative damage can extend lifespan, which depends on the transcription factor DAF-16/FOXO (B. Ayuda-Duran et al., 2019). DAF-16 regulates a FOXO transcription factor in *C.elegans*, which is considered to be a key regulator of lifespan. Therefore, we studied the effects of AOF on mutant strains of *C. elegans* and further explored the expression of its key genes. We found that AOF prolonged the lifespan of *C. elegans via* activation of IIS, particularly by regulating daf-2 and age-1. Normally, IIS is active and phosphorylates DAF-16 *via* AKT and SGK kinases, thereby inhibiting the accumulation of DAF-16 in the nucleus. However, stress can inhibit IIS-induced phosphorylation and promote the translocation of DAF-16 into the nucleus.To verify that DAF-16 plays a key role in AOF, we found the AOF can increased the mRNA expression of daf-16, and activation *daf-16:GFP(TJ356)* nuclear translocation. Therefore, We tested the lifespans of the *daf-16(mu86) I* mutant*,* the results show that AOF can not extend its the lifespans. Furtherly, SOD-3 is the downstream target of DAF-16, which can be used to verify the SOD-3 are regulated by DAF-16 after AOF treatment. We found that the mRNA level of SOD-3 in wild-type was also significantly increased, and the fluorescence expression of *sod-3: GFP(CF1553*) was enhanced after AOF treatment. Thus we confirmed that AOF can reduce insulin-like signaling pathway and prolong the lifespan of *C. elegans* by the DAF-16*.*

Except for DAF-16 signaling, SKN-1 plays a key role in the stress and longevity phenotypes associated with reduced IIS pathway (Jiang et al., 2021). SKN-1 pathway was also involved in AOF-induced lifespan extension of *C. elegans*. We found that AOF promoted SKN-1 expression, also the fluorescence expression of *gst-4::GFP(CL2166)* was enhanced after AOF treatment, which the GST-4 is the downstream target of SKN-1, and the extension of lifespan was abolished in a *skn-1(zu135)* mutant. This suggests that AOF extends the lifespan of *C.elegans via* IIS and SKN-1 pathways.

## 5 Conclusion

This study showed that AOF could mitigate age-related phenotypes, improve resistance against thermal and oxidative stress, and prolong lifespan in *C.elegans*. AOF diminished intracellular ROS and MDA and increased the activities of antioxidant enzymes, suggesting that the anti-aging effect of AOF is due to its antioxidant activity. The effect of AOF on life extension is likely mediated through the upregulation of daf-16, skn-1, sod-3 and gst-4, and the downregulation of daf-2 and age-1. AOF also promoted the migration of DAF-16 and SKN-1 into the nucleus. Characterization of mutants in daf-16 and skn-1 suggested that the extended lifespan by AOF was partly due to these genes. In conclusion, AOF appears to promote longevity and stress resistance *via* the IIS and SKN-1 pathways. This study provides new insights into the anti-aging effect of AOF and may inform further studies on the therapeutic effects of AOF.

## Data Availability

The original contributions presented in the study are included in the article/supplementary material, further inquiries can be directed to the corresponding authors.
