# Supplementary figures and images for: Alpiniae oxyphylla fructus extract promotes longevity and stress resistance of C. elegans via DAF-16 and SKN-1
Source: Front Pharmacol. 2022 Nov 24;13:1034515. doi: 10.3389/fphar.2022.1034515 (PMC9730235; doi:10.3389/fphar.2022.1034515)

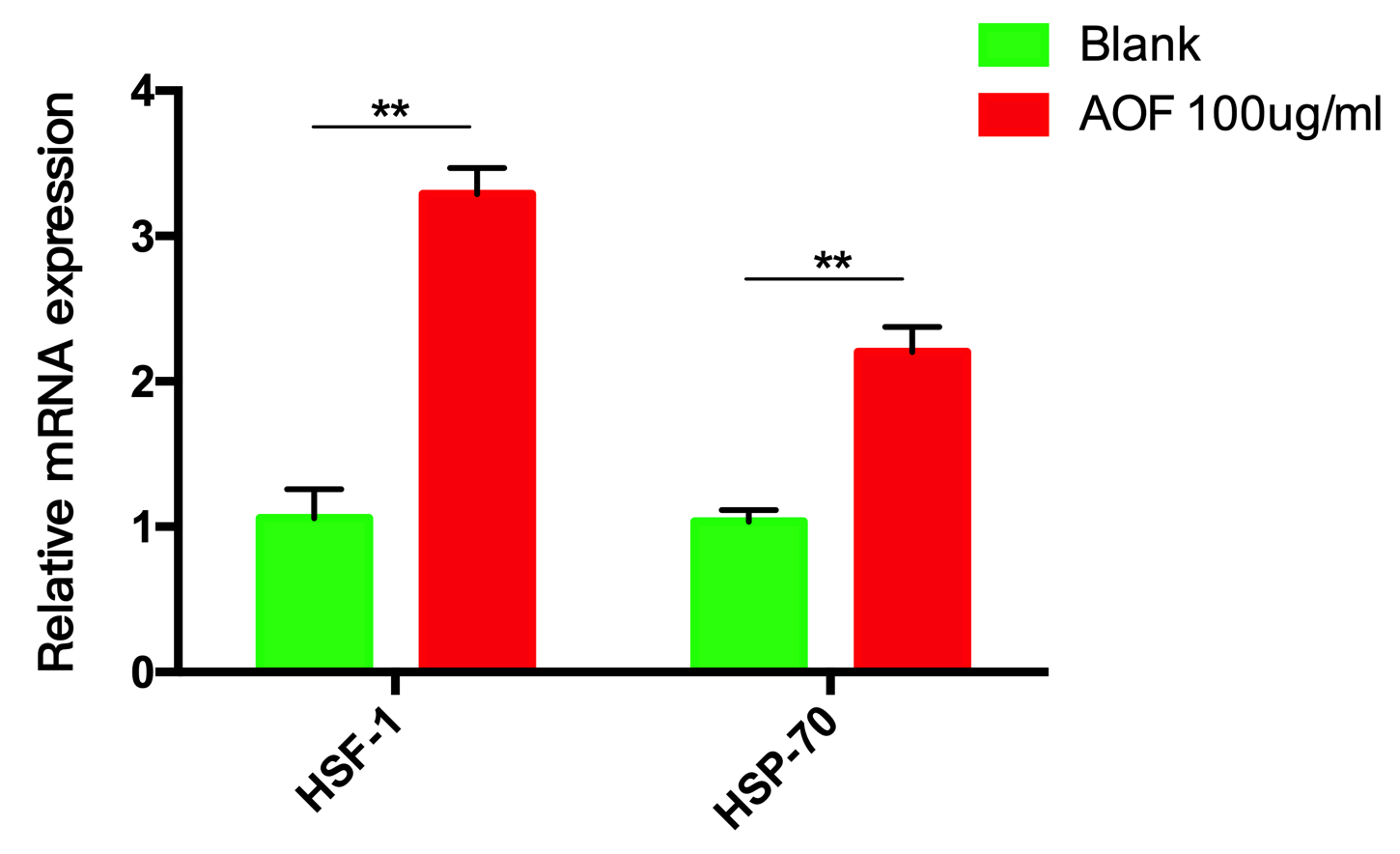

Supplement: Supplementary file 1 [file Image3.TIFF]

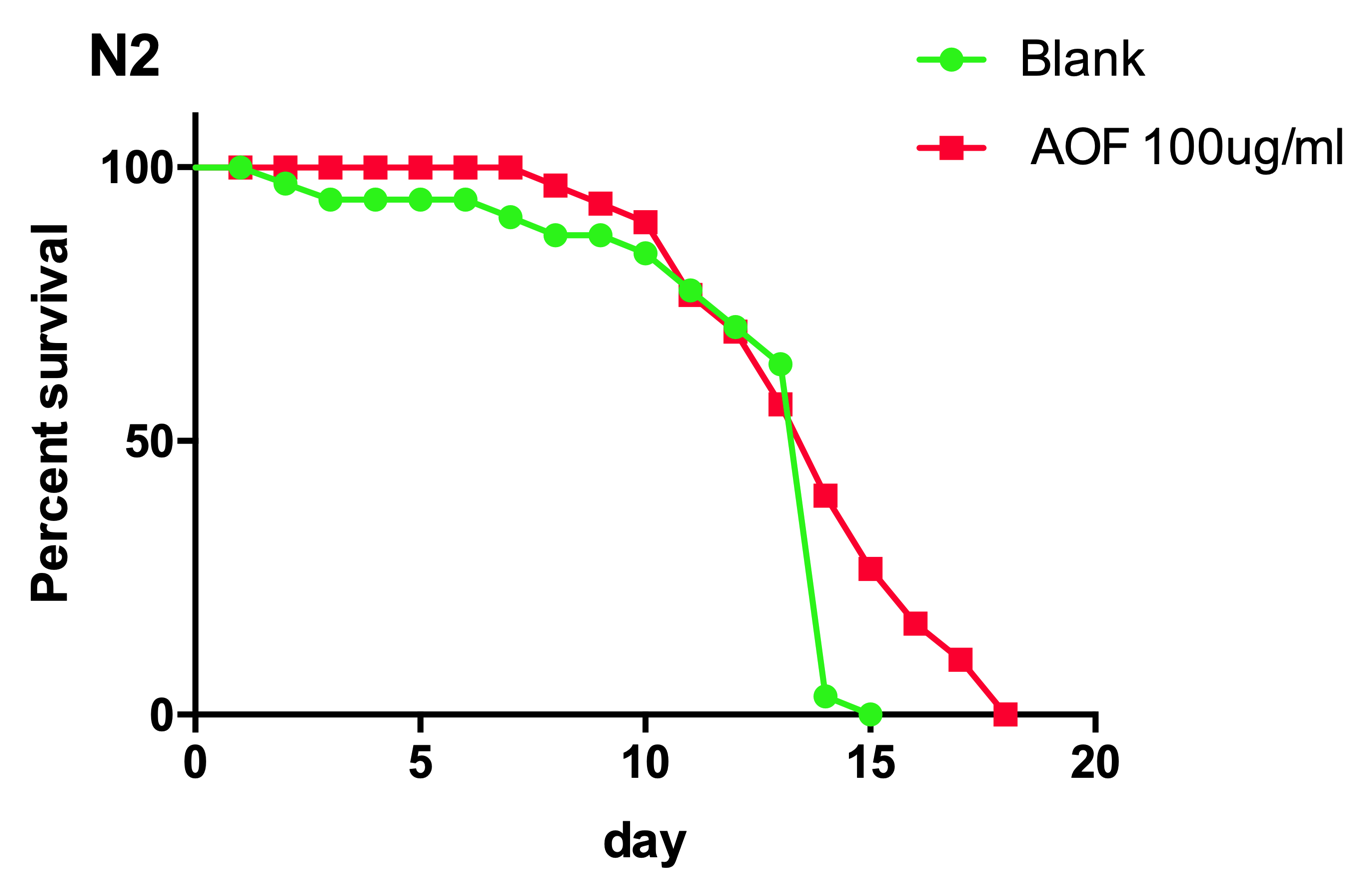

Supplement: Supplementary file 2 [file Image1.TIFF]

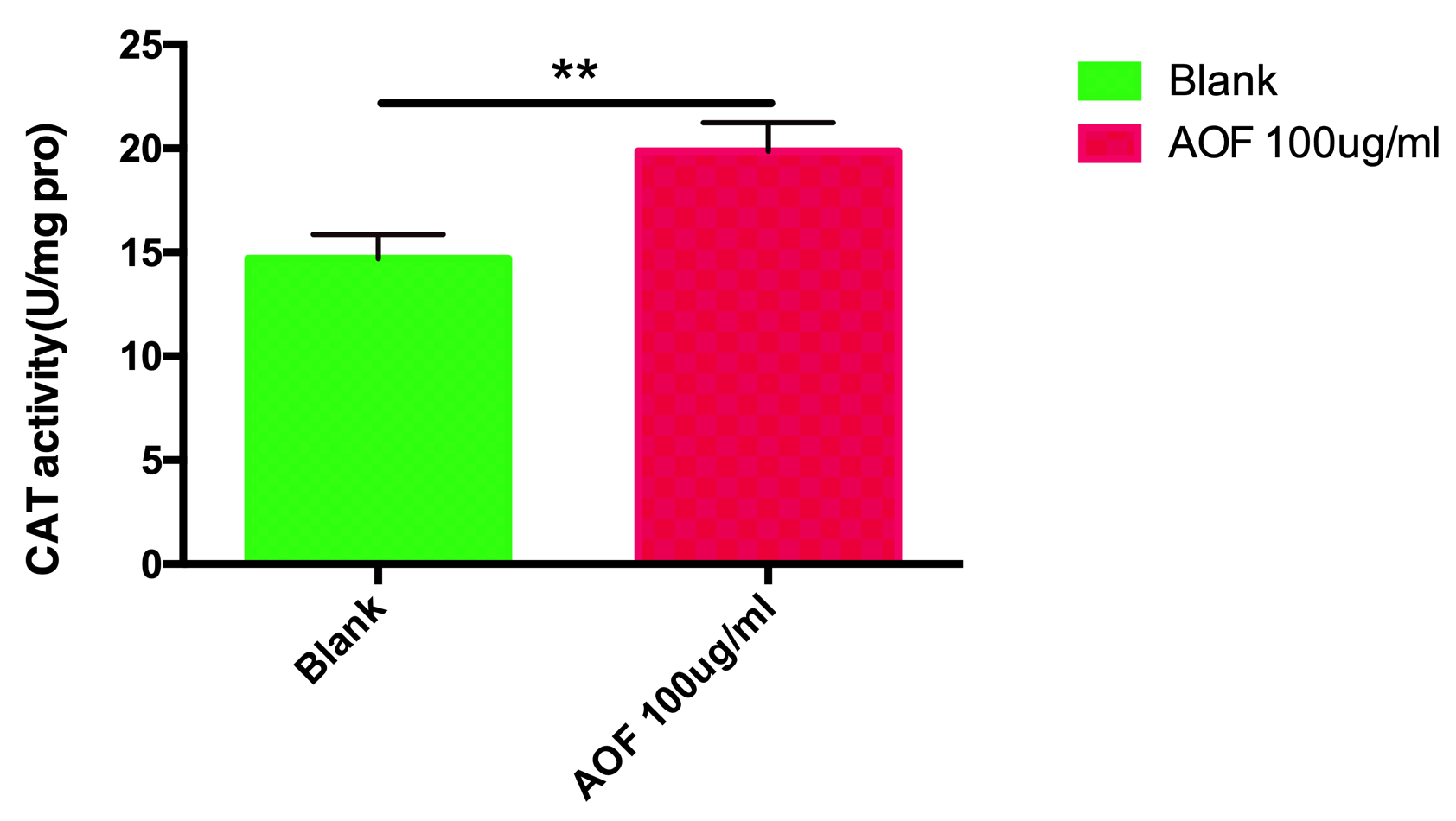

Supplement: Supplementary file 3 [file Image9.TIFF]

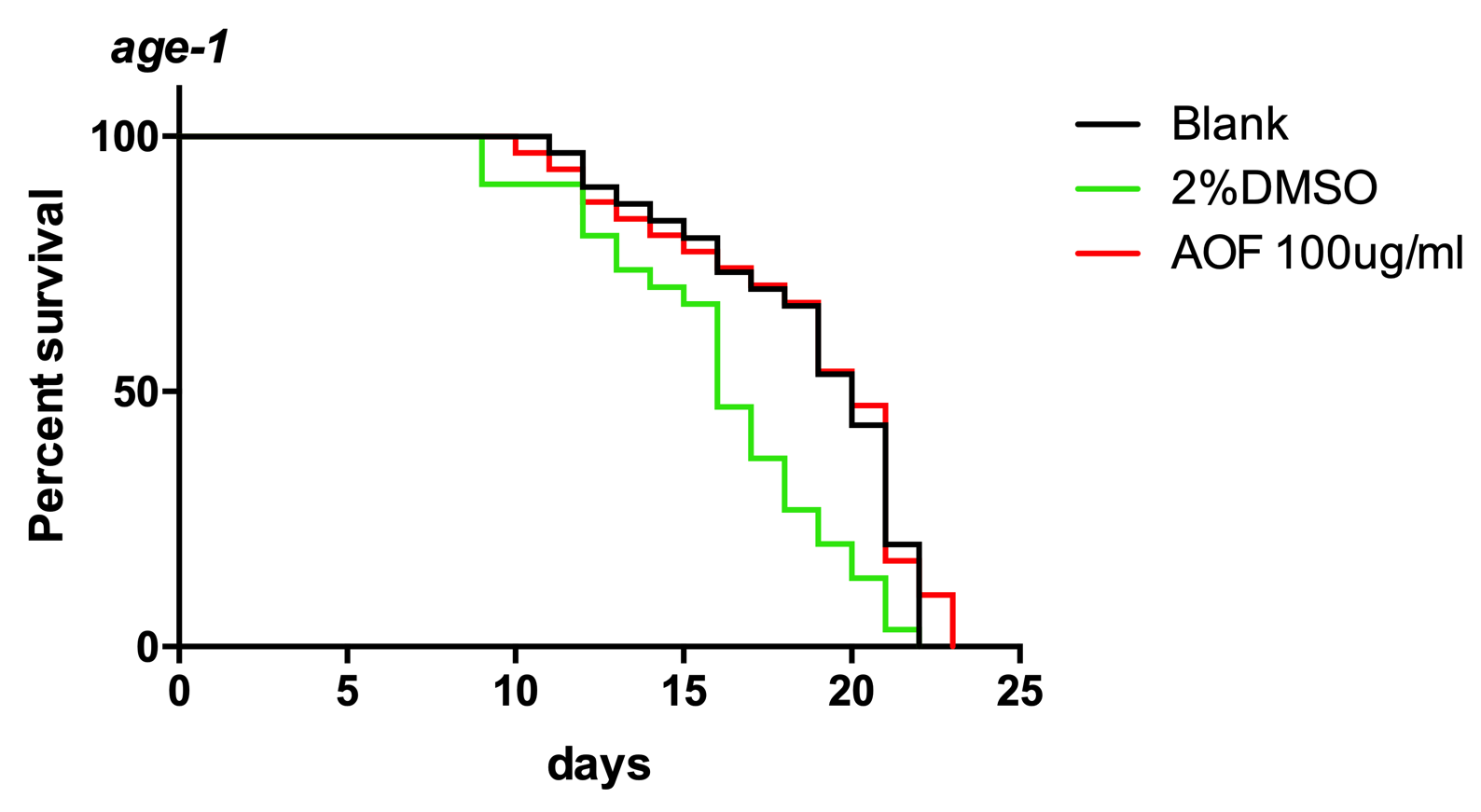

Supplement: Supplementary file 4 [file Image14.TIFF]

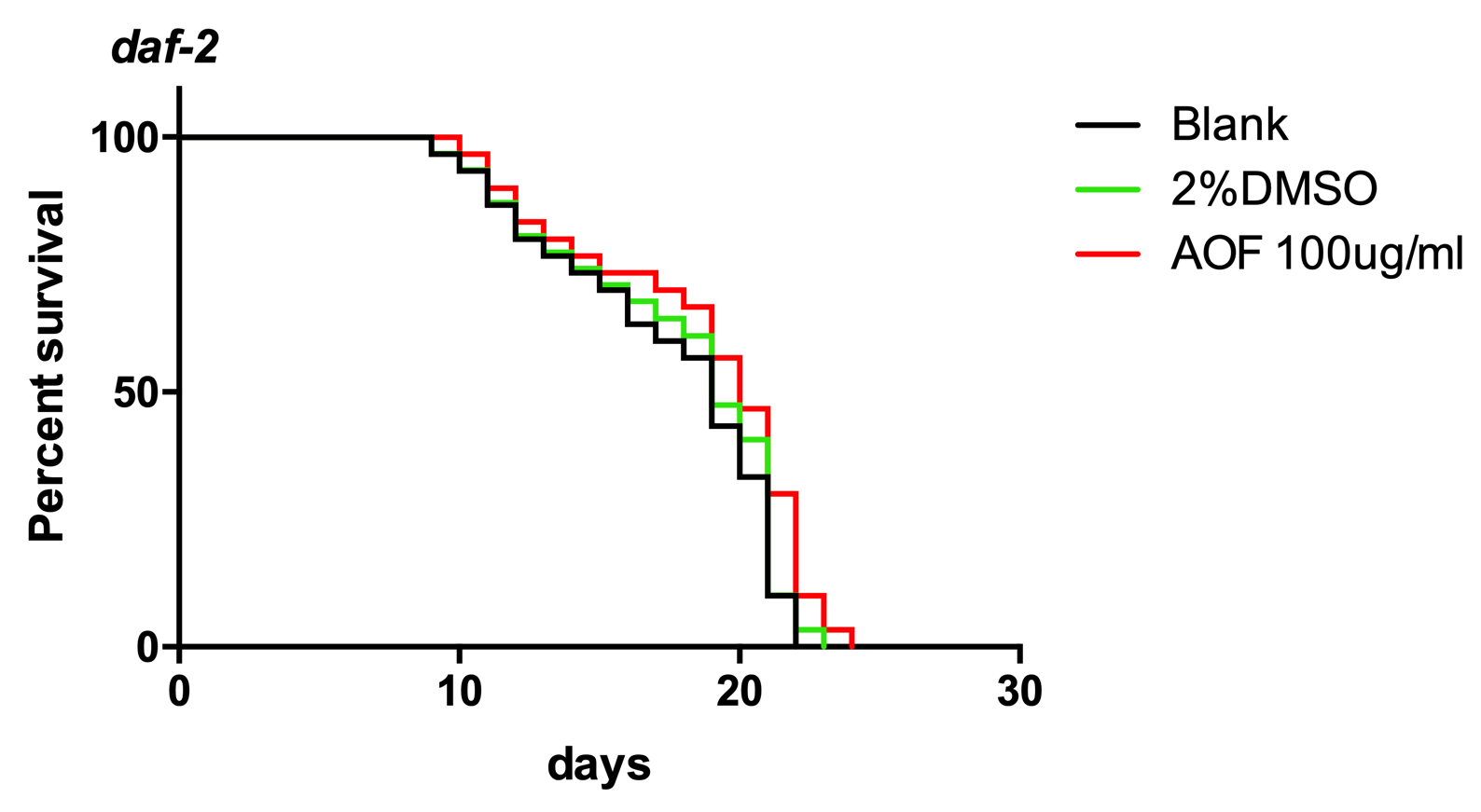

Supplement: Supplementary file 5 [file Image13.TIFF]

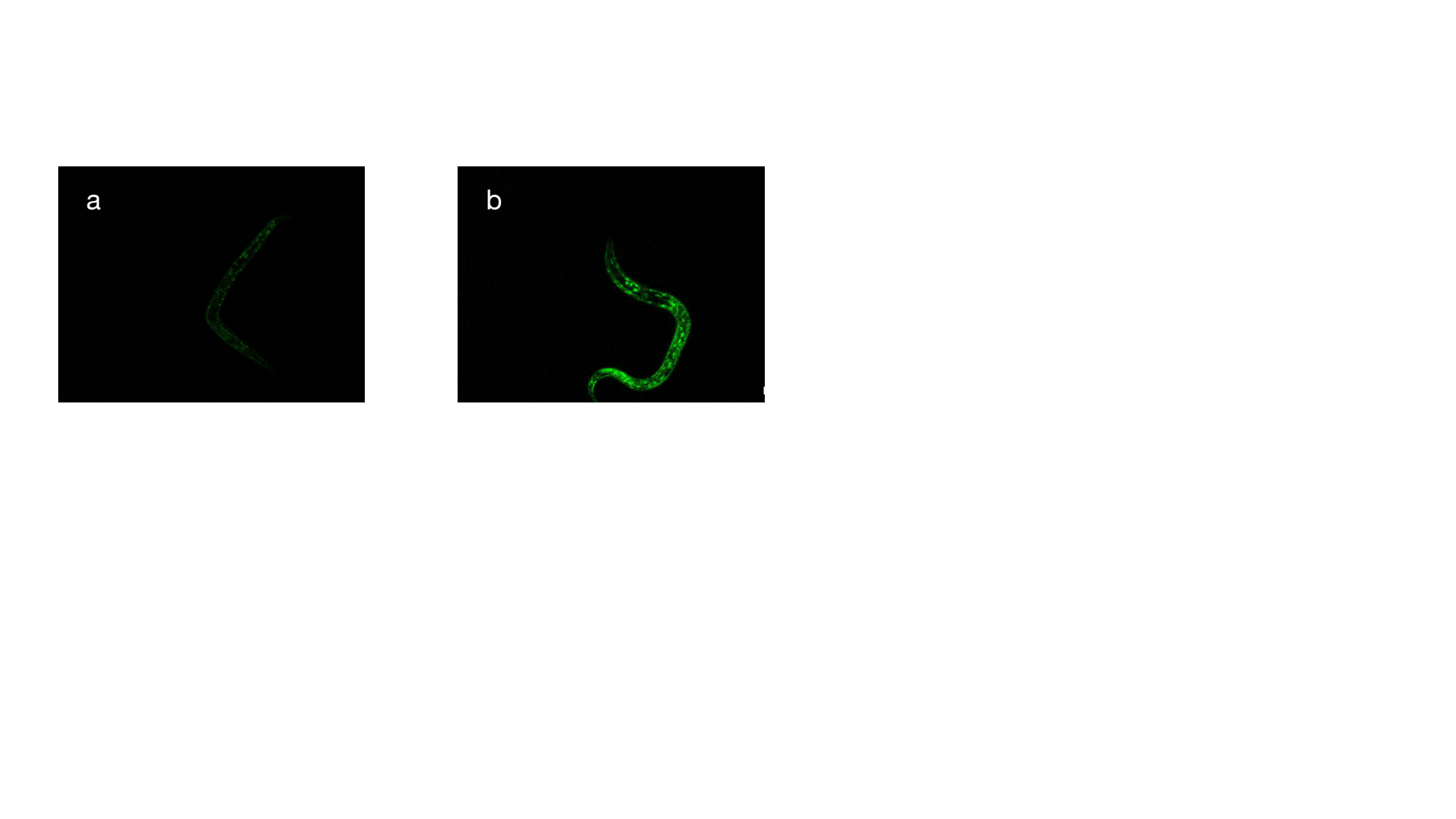

Supplement: Supplementary file 6 [file Image2.TIF]

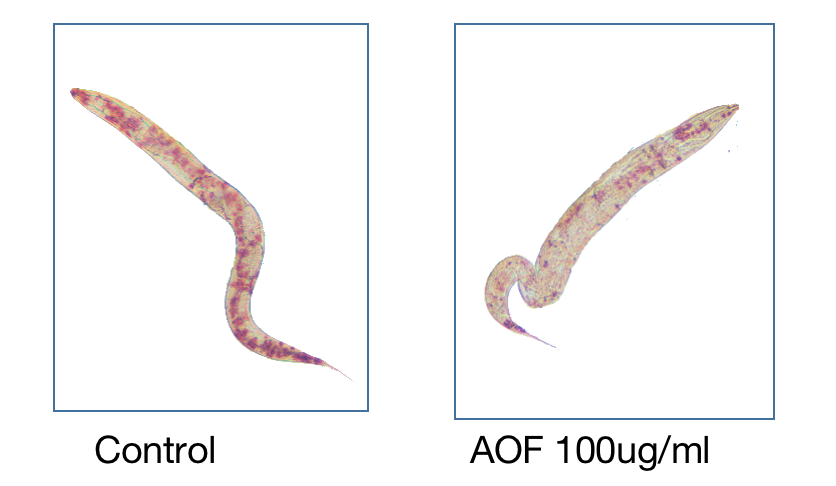

Supplement: Supplementary file 7 [file Image5.PNG]

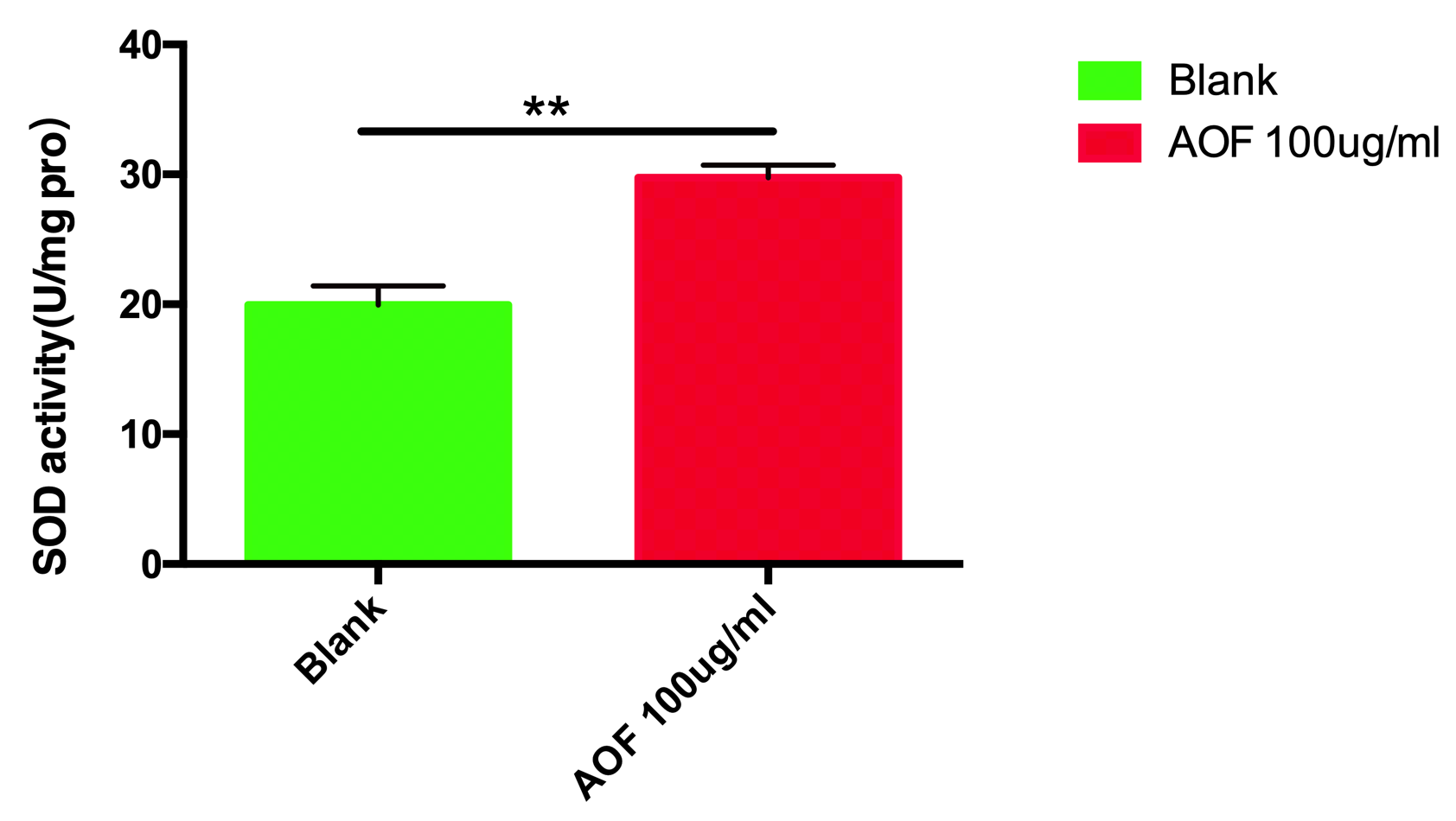

Supplement: Supplementary file 8 [file Image8.TIFF]

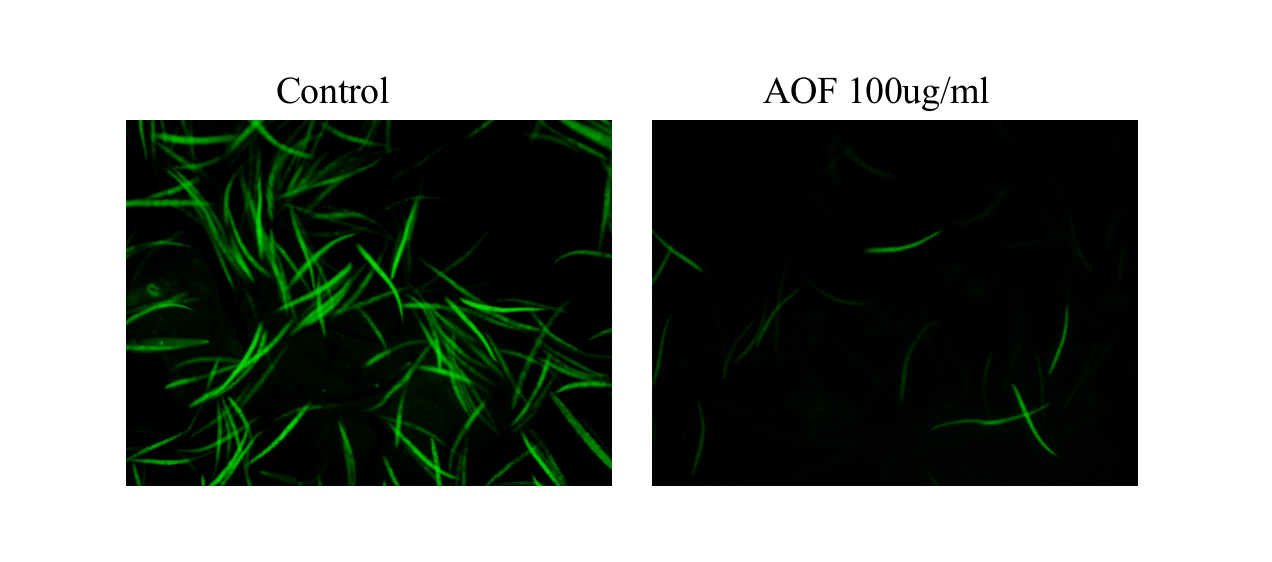

Supplement: Supplementary file 9 [file Image7.PNG]

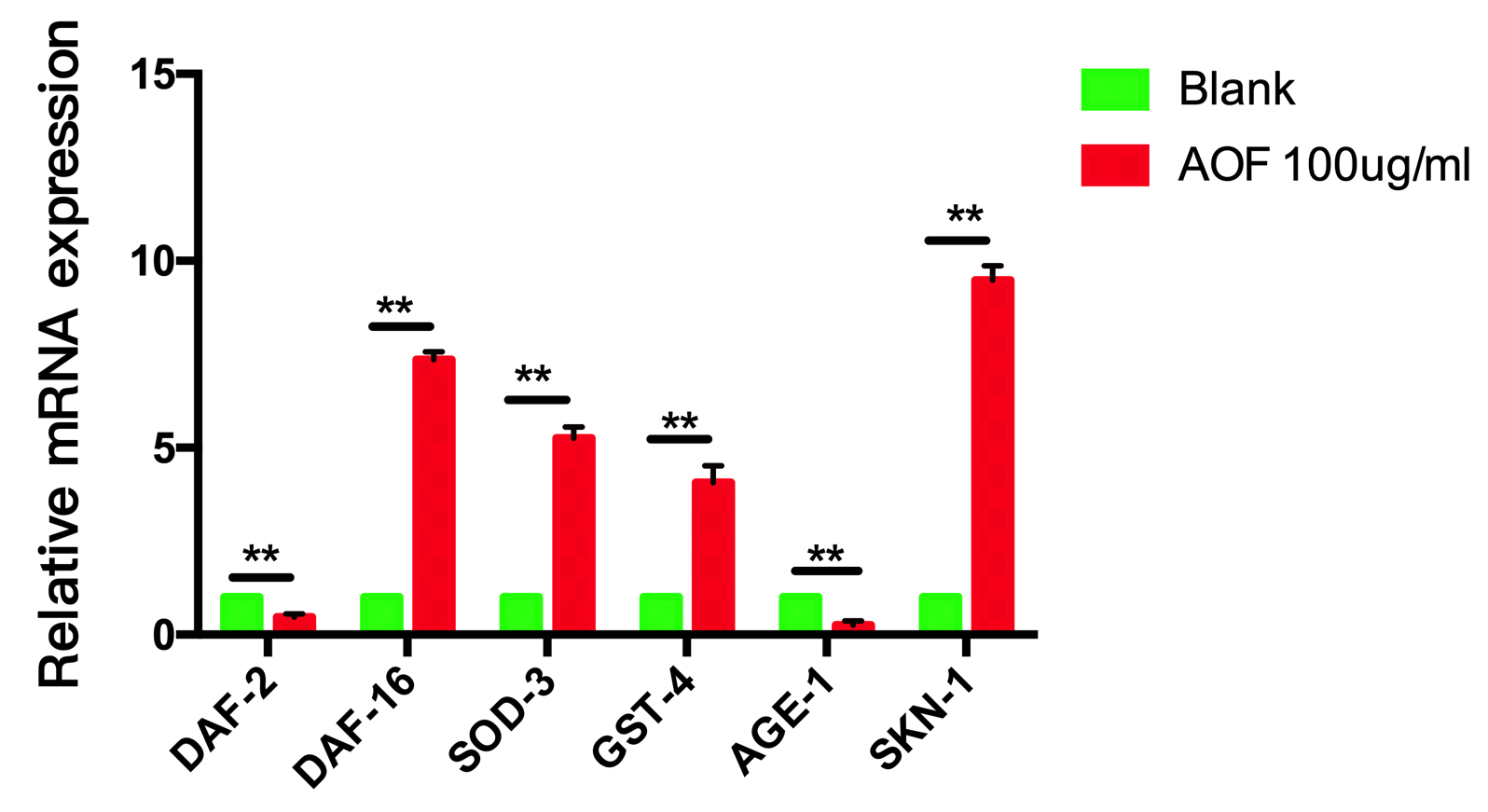

Supplement: Supplementary file 10 [file Image11.TIFF]

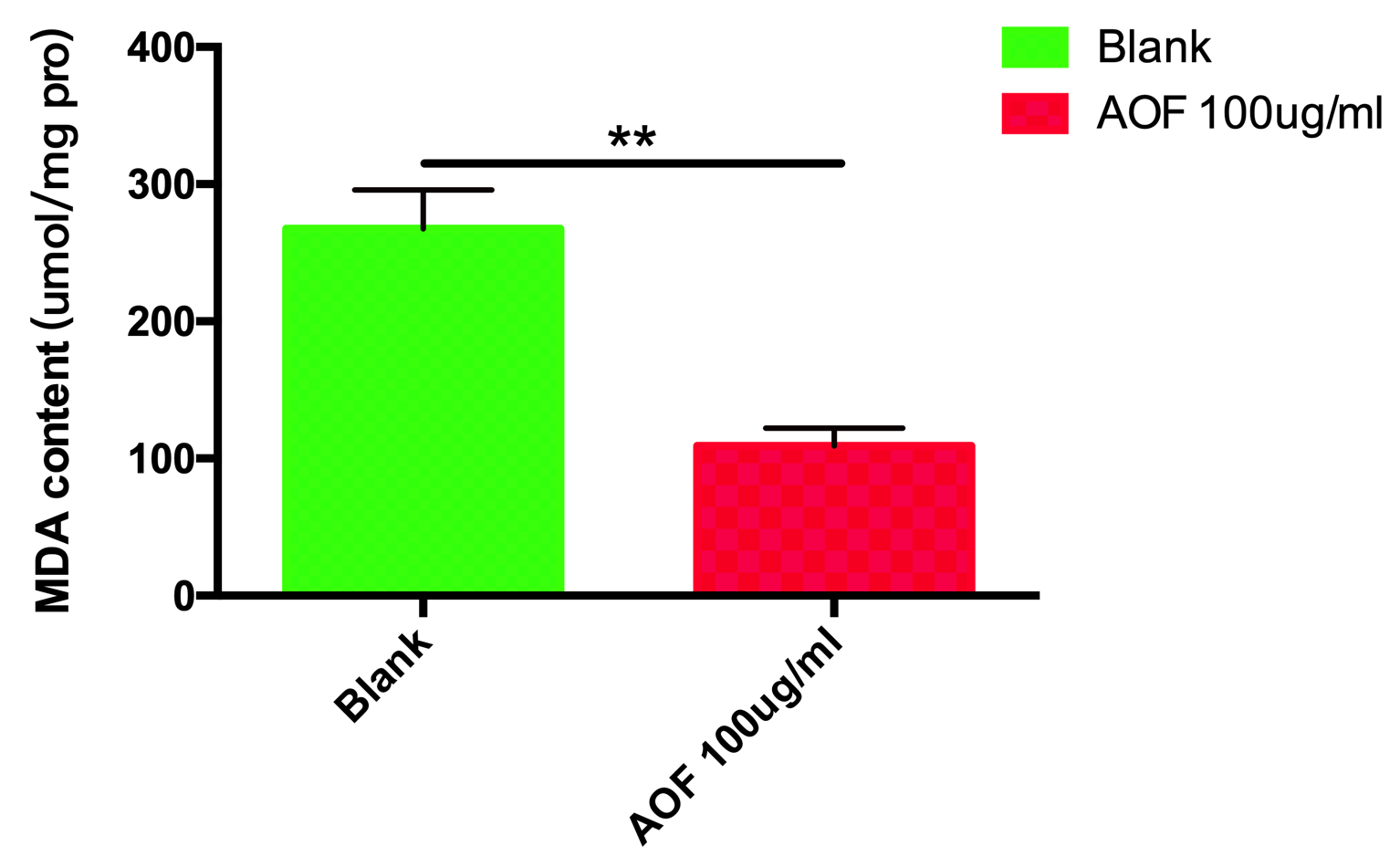

Supplement: Supplementary file 12 [file Image10.TIFF]

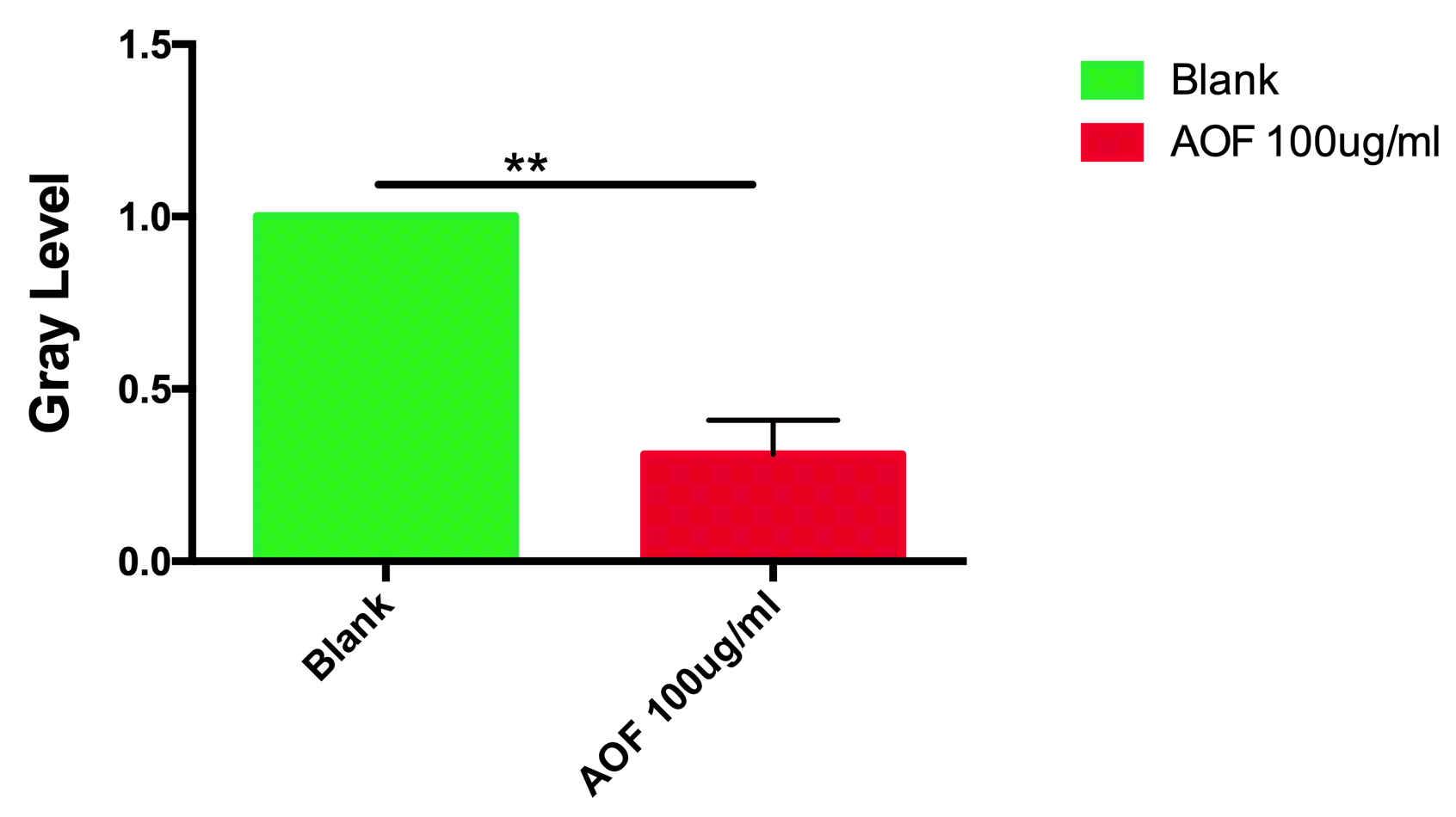

Supplement: Supplementary file 13 [file Image6.TIFF]

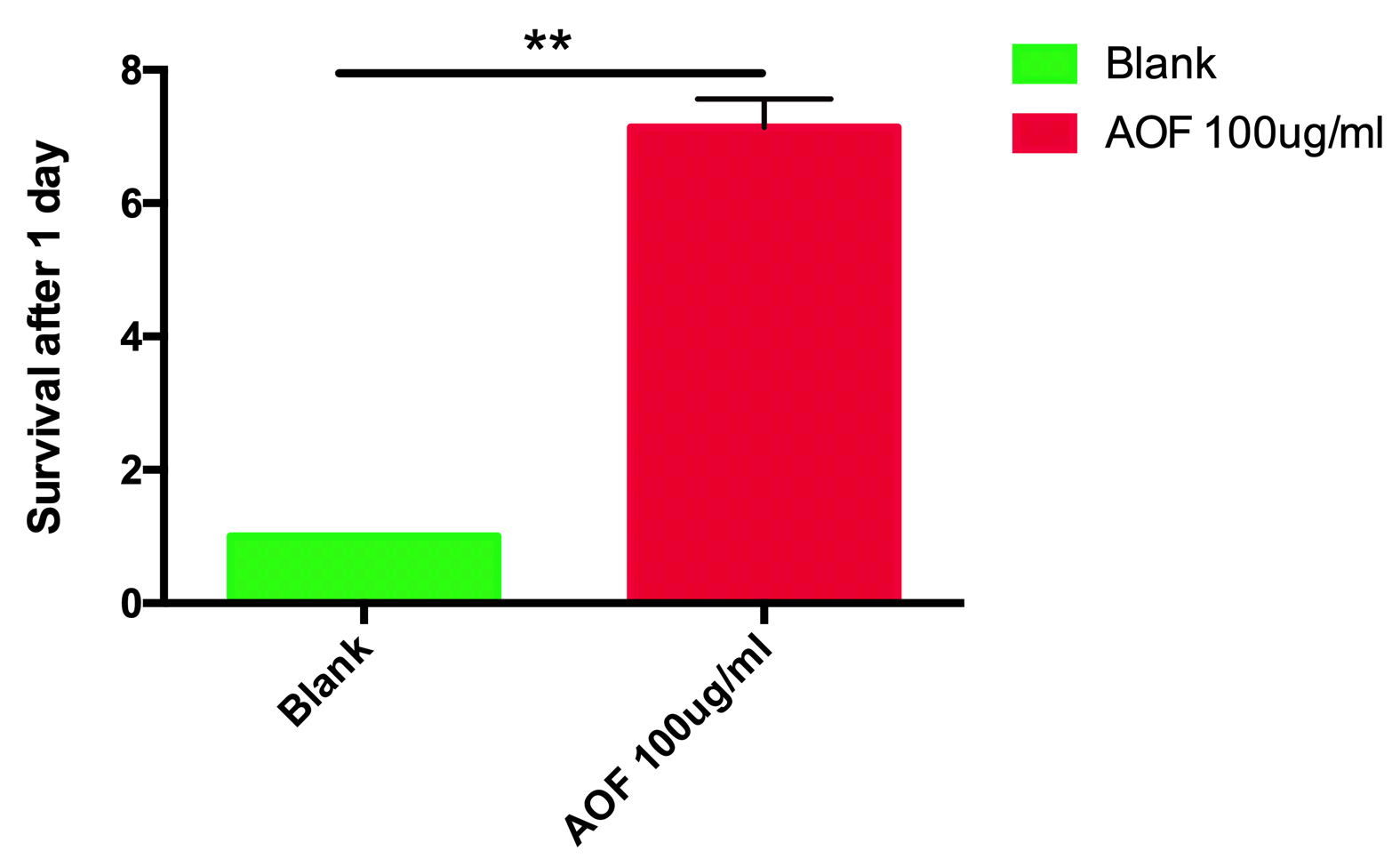

Supplement: Supplementary file 14 [file Image4.TIFF]

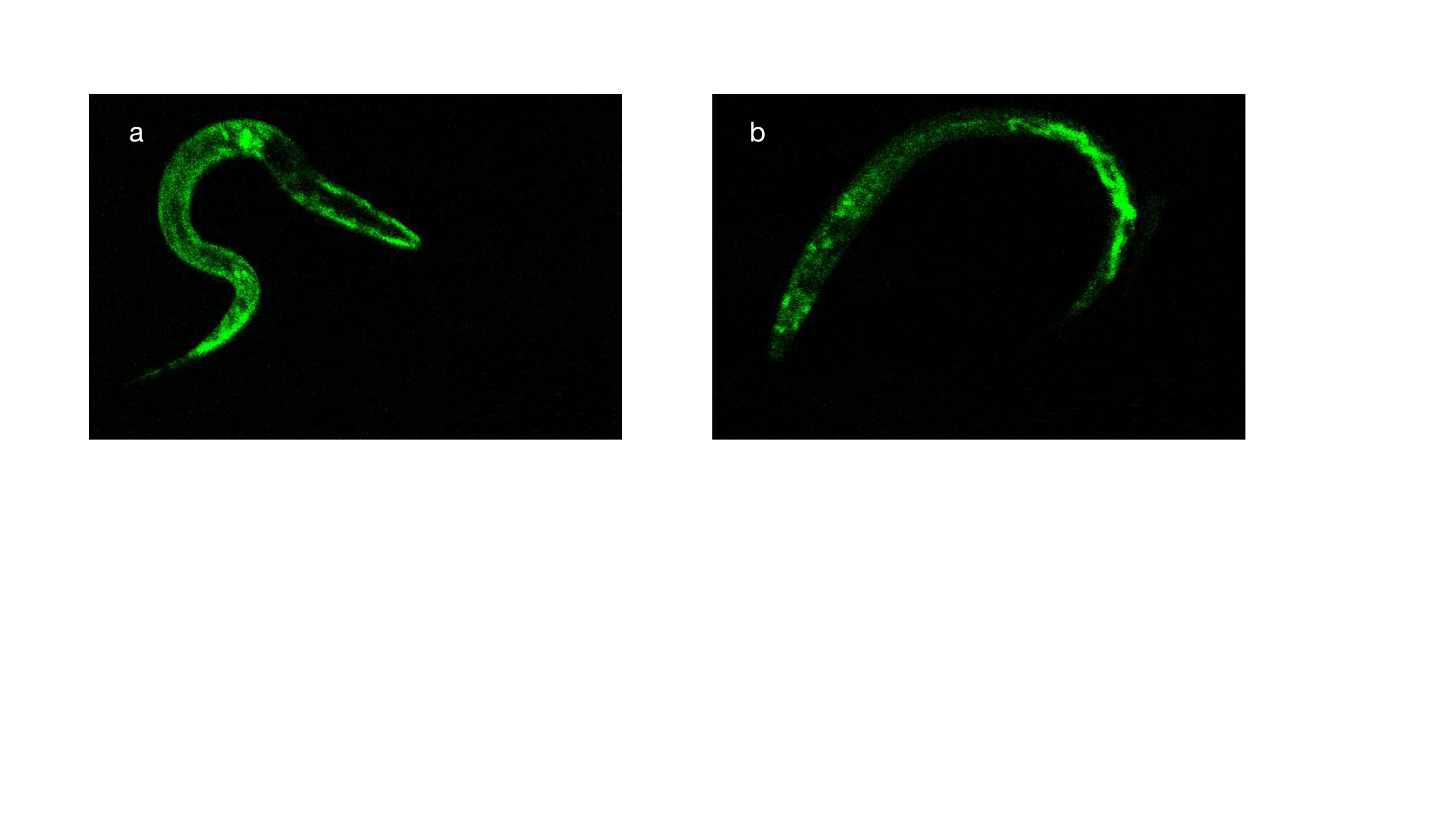

Supplement: Supplementary file 15 [file Image12.TIF]
